# The gut microbiota intervenes in glucose tolerance and inflammation by regulating the biosynthesis of taurodeoxycholic acid and carnosine

**DOI:** 10.3389/fcimb.2024.1423662

**Published:** 2024-08-14

**Authors:** Jianhua Zhen, Yunan Zhang, Yini Li, Yali Zhou, Yanan Cai, Guangrui Huang, Anlong Xu

**Affiliations:** School of Life Sciences, Beijing University of Chinese Medicine, Beijing, China

**Keywords:** diabetes, gut microbiota, impaired glucose tolerance, metabolome, mRNA sequencing, proteome

## Abstract

**Objective:**

This study aims to investigate the pathogenesis of hyperglycemia and its associated vasculopathy using multiomics analyses in diabetes and impaired glucose tolerance, and validate the mechanism using the cell experiments.

**Methods:**

In this study, we conducted a comprehensive analysis of the metagenomic sequencing data of diabetes to explore the key genera related to its occurrence. Subsequently, participants diagnosed with impaired glucose tolerance (IGT), and healthy subjects, were recruited for fecal and blood sample collection. The dysbiosis of the gut microbiota (GM) and its associated metabolites were analyzed using 16S rDNA sequencing and liquid chromatograph mass spectrometry, respectively. The regulation of gene and protein expression was evaluated through mRNA sequencing and data-independent acquisition technology, respectively. The specific mechanism by which GM dysbiosis affects hyperglycemia and its related vasculopathy was investigated using real-time qPCR, Western blotting, and enzyme-linked immunosorbent assay techniques in HepG2 cells and neutrophils.

**Results:**

Based on the published data, the key alterable genera in the GM associated with diabetes were identified as *Blautia*, *Lactobacillus*, *Bacteroides*, *Prevotella*, *Faecalibacterium*, *Bifidobacterium*, *Ruminococcus*, *Clostridium*, and *Lachnoclostridium*. The related metabolic pathways were identified as cholate degradation and L-histidine biosynthesis. Noteworthy, *Blautia* and *Faecalibacterium* displayed similar alterations in patients with IGT compared to those observed in patients with diabetes, and the GM metabolites, tauroursodeoxycholic acid (TUDCA) and carnosine (CARN, a downstream metabolite of histidine and alanine) were both found to be decreased, which in turn regulated the expression of proteins in plasma and mRNAs in neutrophils. Subsequent experiments focused on insulin-like growth factor-binding protein 3 and interleukin-6 due to their impact on blood glucose regulation and associated vascular inflammation. Both proteins were found to be suppressed by TUDCA and CARN in HepG2 cells and neutrophils.

**Conclusion:**

Dysbiosis of the GM occurred throughout the entire progression from IGT to diabetes, characterized by an increase in *Blautia* and a decrease in *Faecalibacterium*, leading to reduced levels of TUDCA and CARN, which alleviated their inhibition on the expression of insulin-like growth factor-binding protein 3 and interleukin-6, contributing to the development of hyperglycemia and associated vasculopathy.

## Graphical Abstract

Baesd on the results of metagenomic data in diabetes and the multiomics analyses in impaired glucose tolerance, we found that decreased CARN and TUDCA, the metabolites derived from GM, reduced the inhibition on the synthesis of IGFBP-3 and the expression of IL-6 to result in hyperglycemia and related vasculopathy.

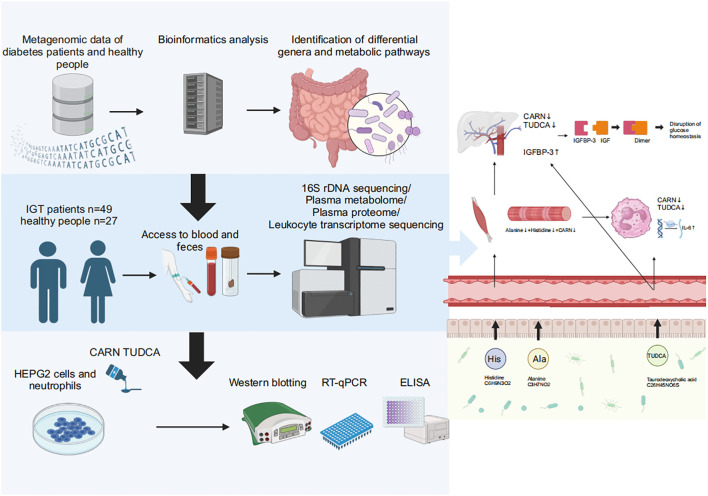

## Introduction

Diabetes is not merely an isolated condition; it is intricately linked with various metabolic disorders and systemic inflammation, leading to complications in multiple organs, including the cardiovascular and renal systems. Given the rising global prevalence of diabetes, the identification of early biomarkers and an understanding of the pathological mechanisms are particularly crucial. However, the progression from a healthy state to diabetes may take several years or even more than a decade, and this individualized progression can be observed in clinical settings (Tabak et al., 2012; Tuso, 2014; Knight et al., 2017). During this period, the oral glucose tolerance test reveals abnormal indices indicating a decreased secretory function of the islets of Langerhans (Schwabe and Jobin, 2013; Poutahidis and Erdman, 2016; Heintz-Buschart and Wilmes, 2018). Furthermore, evidence of corresponding vascular damage has been observed (Yonekura et al., 2005). Consequently, impaired glucose tolerance (IGT) is defined as a fasting plasma glucose (FPG) level < 7.0 mmol/L, while 2-hour postprandial plasma glucose (2hFPG) falls within the range of 7.8 mmol/L to 11.1 mmol/L (Gilbert et al., 2018). Approximately 37% of patients with IGT progress to diabetes within 4 years (DeFronzo and Abdul-Ghani, 2011). Nevertheless, by employing appropriate treatment or intervention, the progression of IGT can be effectively prolonged or reversed (Mithieux, 2018). This suggests that IGT may serve as a potential target for intervention prior to the onset of diabetes.

Previous studies have revealed the significant impact of the gut microbiota (GM) on the development of diabetes. For instance, patients with type 2 diabetes (T2D) exhibit decreased levels of butyrate-producing bacteria and increased levels of pathogenic bacteria in the GM. Therefore, the reduction in butyrate leads to decreased secretion of glucagon-like peptide-1 and peptide YY in the gut. This, in turn, hinders the insulin response and glucose absorption in muscles and adipose tissues, promoting hyperglycemia (Park et al., 2012; Qin et al., 2012). Furthermore, the predicted function based on the 16S rDNA genes in diabetes showed abnormalities in pathways associated with amino acids, fatty acids, sugars, other metabolites, and energy metabolism (Overmyer et al., 2021; Shen et al., 2022). The correlation between the GM and inflammation has been extensively investigated. It is well known that increased intestinal permeability arises due to defective tight junction proteins caused by insufficient butyrate. Subsequently, pathogenic bacteria and their pathogen-associated molecular patterns, such as lipopolysaccharides (LPS), enter the bloodstream or other organs, inducing local or systemic inflammation. This inflammation serves as the underlying pathology in the development of vasculopathy associated with diabetes (Barrett et al., 2017). In addition, Huijuan Yuan et al. identified a correlation between distal symmetric polyneuropathy and the GM. Their research indicated that patients with diabetes exhibited an impaired intestinal barrier due to decreased levels of short-chain fatty acid-producing bacteria and increased levels of LPS-producing bacteria. This imbalance led to a lower tolerance to antigens and increased systemic inflammation and potentially led to the development of distal symmetric polyneuropathy (Yang et al., 2023). In summary, the GM and its metabolites make significant contributions to the development of diabetes and its chronic complications (Ro et al., 2019).

However, there have been few studies focusing on the overall variation in the GM throughout the progression from IGT to diabetes, and the subsequent changes in the metabolites derived from the GM and their impact on the expression of genes and proteins also remain largely unclear, while which may be crucial in elucidating the pathogenesis of diabetes and its associated chronic complications. This study is dedicated to unveiling the complex interactions between the GM and diabetes, including its precursory states such as IGT. We conducted a comprehensive analysis of published GM metagenomic sequencing data to identify characteristic genera and the related functional pathways in diabetes. Furthermore, we recruited 49 patients with IGT and 27 healthy volunteers (HC) to investigate changes in the GM, GM-derived metabolites, plasma proteins, and gene expression in peripheral leukocytes during IGT using multiomics approaches, and explored the intimate relationships between these changes and the disease progression. Based all above, we intended to clarify the specific role of the GM and its metabolites in the pathogenesis of diabetes and associated chronic complications, particularly how they influence insulin sensitivity and glucose homeostasis in the host through impacting metabolic pathways and inflammatory responses, and these results may pioneer new avenues for the prevention and treatment of hyperglycemia, especially slow down or potentially reverse the progression of diabetes by modulating the composition and function of the GM and/or supplementing the metabolites derived from the GM.

## Materials and methods

### Datamining of published metagenomic sequencing data of diabetes

We obtained metagenome raw data about diabetes from the NCBI (National Center for Biotechnology Information) database (https://www.ncbi.nlm.nih.gov/), and these fastq double-end sequencing files were dehosted using Kneaddata (version 0.6.1) and qulified in FastQC (version 0.11.8). Then, the qualified sequences were merged using Concat (version 1.1.5) to obtain clean reads, and information about the taxonomy and function was acquired by mapping these clean reads to the METACYC (Metabolic Pathways From All Domains of Life) database (https://metacyc.org) using HUMAnN2 (version 2.8.1). The visualization of the above results was achieved in R (version 4.2.2). The beta diversity was visualized in principal component analysis (PCA) based on Bray-Curtis’s distance, while the difference between groups was further shown in the partial least squares discriminant analysis (PLS-DA). The differential genera between groups were distinguished in the Wilcoxon rank-sum test and displayed in barcharts. Heatmaps were used to demonstrate the differential metabolic pathways. Prokka (version 1.14.6)-based annotation was performed after merging the double-ended files using Megahit (version 1.2.9), and the differential expressed genes (DEGs) were presented in the volcano plot.

### Subject recruitment and sample collection

In this study, all patients with IGT were recruited from the Beijing University of Chinese Medicine Third Affiliated Hospital (Beijing, 100029, China) under the diagnostic criteria that FPG was within 6.1–7.0 mmol/L while 2hFPG was within 7.8–11.1 mmol/L. For the patients with IGT recruited in our study, we ensured that they were not taking any medications at all during the last 3 months before enrollment, including anti-diabetic drugs. Unfortunately, those subjects with serious diseases of other systems, such as the circulatory system, hematopoietic system, digestive system, and endocrine system, would be excluded, as would those infected with HBV/HCV/HIV/Treponema pallidum. The female subjects who were pregnant/breastfeeding/planning to be pregnant within 6 months and those subjects who participated in other clinical research were also requested to drop out of this study.

Healthy volunteers with matched genders and ages were enrolled as the HC group from Beijing University of Chinese Medicine (Beijing 102488, China), and their FPGs were within 3.9–6.1 mmol/L, while 2hFPGs were less than 7.8 mmol/L. There were no abnormal indices in the physical examination, including routine blood/urine tests, liver and kidney function, blood lipids, and glycosylated hemoglobin. No medication history in the past 3 weeks before inclusion, especially drugs regulating gastrointestinal motility, gastric acidity inhibitors, microecological agents and immunosuppressants.

According to the approved protocol, fecal and blood samples were collected in the hospital and transferred to the laboratory within 2 hours (fecal samples: on dry ice; blood samples: on ice). In the laboratory, the collected blood samples in 10 ml EDTA tubes (BD, New York, USA) were centrifuged at 500 G for 30 min, and the supernatant plasma sample was transferred into another Eppendorf tube. Five milliliters of erythrocyte lysis buffer (Solarbio, Beijing, China) was added to the remaining precipitated cells, and the tube was placed on ice for 15 min. Then, the cells were centrifuged at 500 G for 15 min, and the supernatant was removed. The above lysis step was repeated three or four times until there was no visible red precipitate after centrifugation. Added 2 ml of 1 × PBS solution to the precipitated cells, blowed the mixture gently and centrifulgated with 500 G for 30 min once more. After that, the supernatant was transferred, and 1 ml TRIzol reagent (Thermo Fisher Scientific, Waltham, USA) was added to the precipitate, which was the peripheral leukocyte sample used for mRNA sequencing. Fecal samples were stored in fecal collectors and separated into 3 Eppendorf tubes. All samples were stored at -80 °C for the downstream experiments.

### Illumina sequencing of 16S rDNA and bioinformatic analysis

Fecal samples from the IGT and healthy groups were processed for microbial genomic DNA extraction using the PF Mag-Bind Stool DNA Kit (Omega, Norcross, USA). Firstly, grinded 0.5 g of fecal sample with 250 mg magnetic beads and 700 µL sodium lithium xylene (SLX) in a 2 mL tube at 45 Hz for 5 min; then added 70 µL denaturing solution (DS) buffer and incubated at 70 °C for 10 min and at 95 °C for 2 min. After that, centrifuged the sample at 13000 G for 5 min, and collected 500 µL supernatant to mix with 170 µL protein precipitation buffer and 170 µL high throughput recovery (HTR), then incubated in ice for 5 min. Centrifuged again (13000 G, 5min), and combined 450 µL supernatant with 450 µL XP5 buffer and 40 µL magnetic beads, reacted for 8 min and magneted by the frame, discarded the raffinate and washed the beads with XP5 buffer, poly-β-hydroxybutyrate (PHB), and sterile purified water (SPW), successively. Finally, centrifuged the beads for several seconds (13000 rpm) and discarded the raffinate, dried them by airing for 8 min. The genomic DNA from GM in the feces was absorbed on the surface of the beads and was eluted with elution buffer. DNA integrity was checked using 1% agarose gel electrophoresis, and concentration and purity were measured with a NanoDrop2000 (Thermo Fisher Scientific, Waltham, USA). Using the extracted DNA as a template, PCR amplification of the V3-V4 variable region of the 16S rDNA gene was performed. The forward primer 338F (5’-ACTCCTACGGGAGGCAGCAG-3’) and the reverse primer 806R (5’-GGACTACHVGGGTWTCTAAT-3’), both containing barcode sequences, were used. The amplification profiles were constructed according to previous studies (Lawley and Tannock, 2017; Zafeiropoulos et al., 2020). Quality control of the paired-end raw sequencing reads was performed using fastp (https://github.com/OpenGene/fastp, version 0.19.6). The reads were then assembled using Fast Length Adjustment of Short Reads (FLASH; http://www.cbcb.umd.edu/software/flash, version 1.2.11). Operational taxonomic unit (OTU) clustering of the sequences was conducted using UPARSE (http://drive5.com/uparse/, version 11) at 97% similarity. The representative sequence of each OTU was assigned to the SLIVA 138/16S rDNA bacteria database (https://www.arb-silva.de/) to obtain taxonomic information.

The bacterial community richness, evenness and diversity were assessed with alpha indexes, for example, the Sobs, Shannon, Simpson, ACE and Chao indexes, and the beta diversity was visualized in principal coordinate analysis (PCoA) and Adonis analysis based on Bray-Curtis’s distance, while the difference between groups was further shown in the PLS-DA. The function of the GM was predicted by assigning 16S rDNA genes to the Kyoto Encyclopedia of Genes and Genomes (KEGG) database (https://www.kegg.jp). The differential genera/KEGG orthologies (KOs) were distinguished with the Wilcoxon rank-sum test and linear discriminant analysis effect size (LEfSe) analysis. Afterward, the area under the curve (AUC) in the receiver operating characteristic (ROC) curve was used to evaluate the diagnostic value of the key differential genera, and the related pathways were obtained by mapping the differential KOs to the KEGG database. Spearman’s correlation coefficient was used to assess the relationship between genera and the metabolites in the plasma.

### Construction of metabolite profiles in plasma and bioinformatic analysis

Plasma samples were thawed at 4°C, and 100 μL samples were transferred into a 1.5 mL centrifuge tube. Next, 400 μL extraction solution (methanol: acetonitrile, 1:1, V: V) with 0.02 mg/mL L-2-chlorophenylalanine was added to the tube, which was vortexed for 30 s and then extracted with ultrasound (40 Hz) at 5°C for 30 min. After that, the sample was placed at -20°C for 30 min and centrifuged for 15 min (13000 G, 4°C), and the supernatant was blown with nitrogen until the powder appeared. A 100 μL mixed solution (acetonitrile: water, 1:1, V: V) was used to dissolve the extracted powder, which was vortexed for another 30 s and extracted with ultrasound (40 Hz) at 5°C for another 5 min. The supernatant obtained after centrifugation for 10 min (13000 G, 4°C) was transferred to the injection vial for detection. The quality control sample was a mixture of 20 μL of supernatant from every sample.

The detection of the metabolites in this study used UHPLC-Q Exactive HF-X (Thermo Fisher Scientific, Waltham, USA) and an ACCQUITY UPLC HSS T3 chromatographic column (100 mm × 2.1 mm i.d., 1.8 μm; Waters, Milford, USA); mobile phase A was a water solution containing 5% acetonitrile and 0.1% formic acid, while mobile phase B was a water solution containing 47.5% acetonitrile, 47.5% isopropanol and 0.1% formic acid. The injection volume was 2 μL, and the column temperature was 40°C. The elution procedure was as follows: 0–3.5 min, 100% A, 0.4 mL/min; 3.5–5 min, 75.5% A, 24.5% B, 0.4 mL/min; 5–5.5 min, 65% A, 35% B, 0.4 mL/min; 5.5–7.4 min, 100% B, 0.4 mL/min; 7.4–7.6 min, 100% B, 0.6 mL/min; 7.6–7.8 min, 48.5% A, 51.5% B, 0.6 mL/min; 7.8–9 min, 100% A, 0.5 mL/min; and after 9_th_ min, 100% A, 0.4 mL/min. Electrospray ionization was used to separate the compound further, and the mass spectrum signal was collected in both positive and negative ion modes. The spray voltages were 3500 V (+) and 3500 V (-), and the normalized collision energies were set as 20, 40 and 60 eV. Full scan mode (m/z ranged from 70 to 1050) was performed with the resolution set at 60000 for the full MS scans and 7500 for MS^2^ scans. The MS conditions were a heater temperature of 425°C, capillary temperature of 325°C, sheath gas flow rate of 50 arbitrary units, and aux gas flow rate of 13 arbitrary units. A quality control sample was injected at regular intervals, such as every 5–15 samples, to monitor the test stability.

Original data were processed with Progenesis QI software (Waters, Milford, USA) to obtain the data matrix, including retention time, m/z and peak intensity. First, the metabolites were identified by searching the characteristic peaks in databases, such as The Human Metabolome Database (HMDB; https://hmdb.ca/) and METLIN database (https://metlin.scripps.edu/landing_page.php?pgcontent=mainPage). The differential metabolites were screened based on the criteria of *P* < 0.05 and fold change (FC) > 1.1 or < 0.9 and visualized as a volcano plot, OPLS-DA (orthogonal PLS-DA) and heatmap. The related pathways were obtained by mapping the differential metabolites to the KEGG database.

### Construction of protein profiles in plasma and bioinformatic analysis

Eight M urea solution was added to the frozen plasma sample, and the proteins with low abundances were quantified and enriched according to the instructions of the kits (ProteoMiner™ Protein Enrichment Kit, Bio-Rad, Hercules, USA). Then, triethylammonium bicarbonate buffer (TEAB) was added to a 100 μg sample with a final concentration of 100 mM, as well as tris-(2-carboxyethyl) phosphine with a final concentration of 10 mM, and the mixture was reacted for 60 min at 37°C. Iodoacetamide was added at a final concentration of 40 mM, and the reaction needed 40 min at room temperature in the dark. Centrifugation was performed for 20 min at 10000 G, and the precipitate was redissolved in 100 μL of 100 mM TEAB. The proteins were hydrolyzed using trypsin 1:50, m: m at 37°C to obtain peptide segments, which were dried in a vacuum centrifugal concentrator (Huamei, Taicang, China) and redissolved in a solution containing 1% trifluoroacetic acid. These peptide segments were desalinated with a hydrophilic lipophilic balance and quantified after drying in a vacuum centrifugal concentrator according to the instructions of the kits (Thermo Fisher Scientific, Waltham, USA). The peptide segments were dissolved with loading buffer for the downstream tests.

The detection of the proteins used Vanquish F-Q Exactive HF-X (Thermo Fisher Scientific, Waltham, USA) and a C18 column (75 μm×25 cm, Thermo Fisher Scientific, Waltham, USA). A 2% or 80% acetonitrile solution containing 1% formic acid was used as the mobile A or B phase, respectively. The flow rate was 300 nL/min, and the procedure was as follows: 0–70 min, 5% B; 70–90 min, 23% B; 90–100 min, 29% B; 100–102 min, 38% B; 102–103 min, 48% B; 103–120 min, 100% B. The scan mode ranged from 300 to 1500 m/z, the dissociation used the higher energy mode, and 40 variable windows were set.

Raw data acquired in Thermo Xcalibur (version 4.0, Thermo Fisher Scientific, Waltham, USA) were retrieved using Proteome Discoverer Software (version 2.4) to construct the digital fingerprints, which were imported into Spectronau (version 16) to select the ion peak, and the quantitative results were obtained based on the calculation of the peak area. The differential proteins were screened based on the criteria of *P* < 0.05 and FC > 1.2 or < 0.83 and visualized as a heatmap. The related pathways were obtained by mapping the differential proteins to the KEGG database.

### Illumina sequencing of mRNA in peripheral leukocytes and bioinformatic analysis

Total RNA in peripheral leukocytes was extracted using TRIzol individually, and the integrity was tested using agarose gel electrophoresis. The mRNA sequencing library for the Illumina HiSeq platform was built according to the instructions of the NEBNext^®^ Ultra™ RNA Library Prep Kit (Illumina, California, USA). Clean reads were obtained after filtering and GC distribution checking, which were assigned to the genome using HISAT2 software (https://ccb.jhu.edu/software/hisat2/manual.shtml#usage, version 2.2.1). The quantitative analysis of the genome was performed in subread software (http://subread.sourceforge.net/, version 2.0.1), and the DEGs needed to satisfy the criterion of |log_2_FC| ≥ 1 and *P* < 0.05, which was visualized as a heatmap. The related pathways were identified by mapping the DEGs to the KEGG database.

### Purification and isolation of peritoneal neutrophils from mice

Ten percent peptone (Sigma, Saint Louis, USA) was injected twice into the peritoneum of 6- to 8-week-old male C57BL/6N mice within 12 h, with a dosage of 1 mL every time. After 3 h of the second injection, the mice were killed, the skin was separated from the peritoneal wall under aseptic conditions, 1640 medium (Sigma, Saint Louis, USA) containing 10% fetal bovine serum (FBS, Sigma, Saint Louis, USA) was injected intraperitoneally, and the mice were vibrated for 5 min to obtain abdominal lavage fluid. This fluid was transferred to a centrifuge tube after filtering with a membrane of 70 μm (Corning, Corning, USA) and centrifuged for 10 min (1500 rpm) to obtain sediments. The cells were then resuspended in 1640 medium containing 10% FBS gently. Percolls with different densities (70.2% and 54.8%, Sigma, Saint Louis, USA) were mixed to dissociate the different cells. One milliliter of abdominal cell suspension was added to the separation liquid and centrifuged for 30 min (500 G), and then the neutrophils were placed between the two densities. The neutrophils were transferred to a new centrifuge tube, 1× PBS (1:1, V:V) was added, the cells were mixed well and centrifuged for 10 min (1000 rpm), and the supernatant was removed. The above centrifugation was repeated once more, and the neutrophils were resuspended in 1×PBS (1:1, V:V) and cultured at 37°C in a 5% CO2 incubator. Then, 100 ml of PBS solution with cells was added to an Eppendorf tube, to which 10 μL of Ly6g antibody (Abcam, Cambridge, UK) was added and left on ice for 40 min, and the purity of the cells was examined by fluorescence activated cell sorting (**Supplementary Figure 1**).

### Cell culture

HepG2 cells (ATCC, Rockefeller, USA) and neutrophils from the abdominal cavity of mice (C57BL/6N, Beijing SYXK (Jing) 2017–0033, Vitalriver, Beijing, China) were cultured to explore the effect of sodium taurodeoxycholate (TUDCA; YUANYE, Shanghai, China) and carnosine (CARN; Sigma, Saint Louis, USA) on the organism as GM metabolites. Cellular drug delivery concentration determined by the CCK8 (BIORIGIN, Beijing, China) assay. In the culture of HepG2 cells, the intervening concentration of TUDCA was 0.2 mmol/L, 0.4 mmol/L, and 0.8 mmol/L, while CARN was given as 50 mmol/L, 100 mmol/L and 150 mmol/L, and the supernatant and the cells were collected for western blotting and real-time qPCR (RT-qPCR) after 24 h. In the culture of neutrophils, LPS (1 μg/mL, Sigma, Saint Louis, USA) was used to induce the secretion of IL-6 (interleukin-6), and TUDCA was given as 0.2 mmol/L, 0.4 mmol/L, and 0.6 mmol/L, while CARN was given as 10 mmol/L, 20 mmol/L and 40 mmol/L. The supernatant and the cells were collected for enzyme-linked immunosorbent assay (ELISA) and RT-qPCR after 4 h.

### RT-qPCR

RNA was extracted from cells with an RNA extraction kit (Accurate Biology, Changsha, China). Reverse transcription was conducted using a reverse transcription kit (Accurate Biology, Changsha, China), with reaction conditions consisting of a 15-min pre-treatment at 37°C, a 5-sec reaction at 85°C, and then a maintainment at 4°C. RT-qPCR was performed using a Bio-Rad CFX96 Real-Time PCR System (Bio-Rad, Hercules, USA). The PCR procedure followed standard thermocycling parameters: an initial denaturation at 95°C for 30 min, followed by 40 cycles that including a 5-sec reaction at 95°C and a 30-sec reaction at 60°C in each cycle. The primers were as follows: insulin-like growth factor binding protein-3 (IGFBP-3; 5’-AGAGCACAGATACCCAGAACT-3’, 5’-GGTGATTCAGTGTGTCTTCCATT -3’) and IL-6 (5’-CTGCAAGAGACTTCCATCCAG-3’, 5’-AGTGGTATAGACAGGTCTGTTGG -3’).

### Western blotting and ELISA

Protein extraction was performed using RIPA buffer (Salorbnio, Beijing, China) at 4°C for 30 min, and then centrifugation was conducted at 4°C, 14000 G for 20 min to collect the supernatant, which was used to detect the protein content for quantitative analysis with a Pierce™ BCA protein quantification kit (Thermo Fisher Scientific, Waltham, USA). IGFBP-3 was determined through western blotting (Abcam, Cambridge, UK), while interleukin-17C (IL-17C) and IL-6 were determined through ELISA (BIORIGIN, Beijing, China; CLOUD-CLONE CORP, Wuhan, China).

### Statistical analysis

Statistical calculations were performed using SPSS software (version 25.0, SPSS Inc., Chicago, USA). The normalization of distribution was assessed using the Kolmogorov-Smirnov or Shapiro-Wilk test, and then the independent sample t test or Mann-Whitney U test was used for the analysis of variables. The chi-square test was used to analyze the counting data. A significant difference was declared at *P* < 0.05.

## Results

### Gut metagenome analysis of patients with T2D

**Figure 1:**
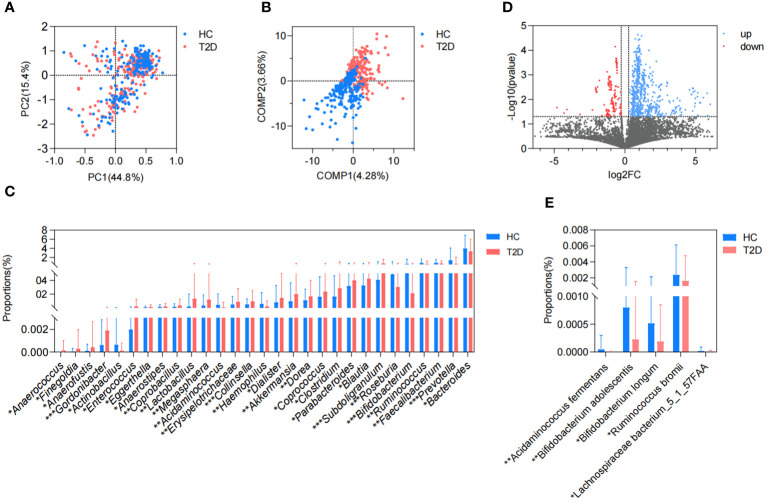
Difference in GM metagenome between patients with T2D and healthy volunteers. **(A)** Beta diversity based on Bray-Curtis distance in PCA. **(B)** PLS-DA. **(C)** Differential genera in the Wilcoxon rank-sum test. **(D)** Volcano plots of the GM gene profiles in patients with T2D and HC volunteers. **(E)** Differential species in cholate degradation and L-histidine biosynthesis. HC, healthy control; T2D, type 2 diabetes; PC, principal component; COMP, component; GM, gut microbiota; PCA, principal component analysis; PLS-DA, partial least squares discriminant analysis. *P<0.05, **P<0.01, ***P<0.001.

In this study, we collected a total of 465 metagenomic samples related to GM, which comprised 225 samples from patients with T2D and 240 samples from healthy volunteers. Subsequently, all the dehosted fastq files met the quality control criteria and were eligible for downstream analysis. PCA based on the Bray-Curtis distance showed no discernible distinction between the T2D and HC groups. (**Figure 1A**), while the separation between groups was magnified and displayed in the PLS-DA (**Figure 1B**), and 29 differential genera were screened in the Wilcoxon runk-sum test, including *Faecalibacterium*, *Blautia*, *Prevotella*, *Bifidobacterium*, *Ruminococcus*, *Subdoligranulum*, *Collinsella*, and *Gordonibacter* (**Figure 1C**; **Supplementary Table 1**). Following functional gene mapping, a total of 7832 genes were identified, including 732 upregulated genes and 189 downregulated genes in patients with T2D (**Figure 1D**). Based on the taxonomic and functional gene annotations, further exploration was conducted on the metabolic pathways and related microbial species (**Supplementary Table 2**). Two pathways, namely, cholate degradation and L-histidine biosynthesis, caught our attention and the related species were presented in **Figures 1E**.

**Figure 2:**
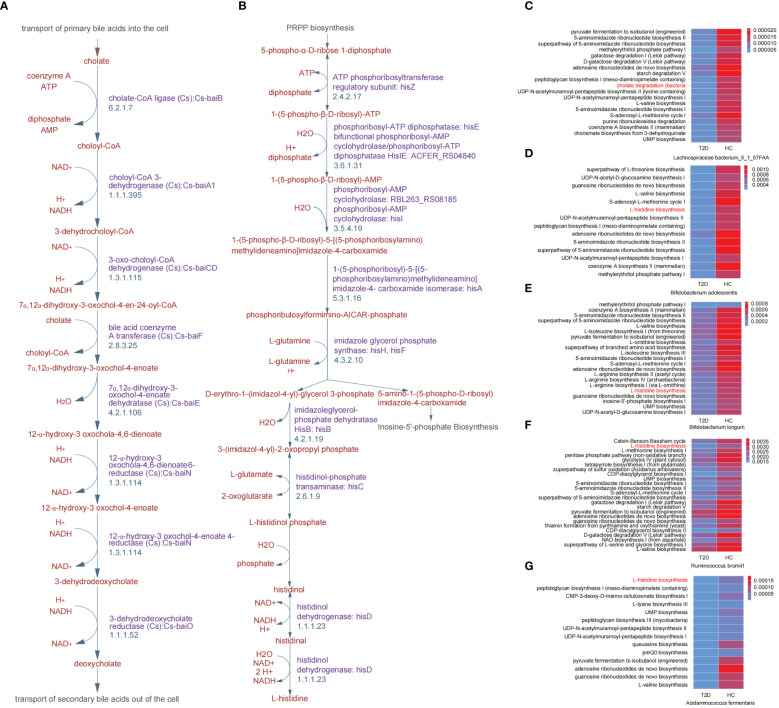
Key differential metabolic pathways between patients with T2D and healthy volunteers. **(A)** Cholate degradation. **(B)** L-histidine biosynthesis. **(C-G)** Overview heatmap of metabolic pathways related to differential species between T2D and HC groups in cholate degradation and L-histidine biosynthesis. HC, healthy control; T2D, type 2 diabetes.

During the cholate degradation process (**Figure 2A**), certain genes played crucial roles, including cholate-CoA ligase (Cs), choloyl-CoA 3-dehydrogenase (Cs), 3-dehydrodeoxycholate reductase (Cs), and 12-α-hydroxy-3 oxochola-4,6-dienoate6-reductase (Cs). These genes, identified as DEGs between the T2D and HC groups, contribute to the conversion of cholate into deoxycholate. The genes encoding transferase, dehydratase and dehydrogenase, for instance, ATP phosphoribosyltransferase, phosphoribosyl-ATP diphosphatase, imidazoleglycerol-phosphate dehydratase HisB, and histidinol dehydrogenase, were all involved in the DEGs between the T2D and HC groups and mapped in the L-histidine biosynthesis process (**Figure 2B**). Meanwhile, 5 gut microbial species were found to be correlated with the above two pathways, and they all exhibited reduced relative abundances in patients with T2D (**Figure 1E**). In addition, the metabolic pathways related to all differential GM were revealed in heatmaps (**Figures 2C–G**; **Supplementary Figure 2**). Except for cholate degradation and L-histidine biosynthesis, the pathways enriched in amino acid metabolism and glycometabolism were highly prominent. These pathways encompassed pyruvate fermentation to isobutanol (engineered), L-valine biosynthesis, the superpathway of L-threonine biosynthesis, L-isoleucine biosynthesis I (from threonine), L-arginine biosynthesis, the citrate cycle (TCA cycle), the pentose phosphate pathway, and glycolysis (**Figures 2C–G**; **Supplementary Figure 2**).

### Clinical characteristics of the IGT and healthy subjects

In total, 49 IGT patients and 27 HC volunteers were included. All baseline details are shown in **Supplementary Table 3**. There was no difference between the IGT and HC groups in terms of sex, age, height, weight, and BMI (body mass index, **Supplementary Tables 4–11**), while there were significant increases in FPG and 2hFPG in the patients with IGT (**Supplementary Tables 5, 9, 11, 13**; FPG: GM, *P* = 0.003, metabolome, *P* = 0.008, proteome, *P* = 0.031, mRNA sequencing, *P* = 0.012; 2hFPG: GM, *P* = 0.009, metabolome, *P* = 0.006, proteome, *P* = 0.004, mRNA sequencing, *P* = 0.009). However, the liver and kidney function indices in the patients with IGT also displayed alterations, such as a decline in the concentration of alanine aminotransferase (ALT; **Supplementary Tables 5, 7, 9, 11**; GM, *P* = 0.005, metabolome, *P* = 0.006, proteome, *P* = 0.015, mRNA sequencing, *P* = 0.017) and rising concentrations of aspartate transaminase (AST), uric acid (UA), creatinine (CREA) and blood urea nitrogen (BUN) (**Supplementary Tables 5, 7, 9, 11**; AST: proteome, *P* = 0.033, mRNA sequencing, *P* = 0.021; UA: GM, *P* = 0.008, metabolome, *P* = 0.007, proteome, *P* = 0.002, mRNA sequencing, *P* = 0.011; CREA: metabolome, *P*=0.044, proteome, *P* = 0.042, mRNA sequencing, *P* = 0.031; BUN: GM, *P* = 0.024, metabolome, *P* = 0.001, proteome, *P* = 0.024, mRNA sequencing, *P* = 0.017). Notably, lipid metabolism exhibited disorder in patients with IGT (**Supplementary Tables 5, 7, 9, 11**), for example, decreases in the concentrations of total cholesterol (TC; proteome, *P*=0.021, mRNA sequencing, *P*=0.023), triglyceride (TG; GM, *P*=0.008, proteome, *P*=0.009, mRNA sequencing, *P*=0.017), high-density lipoprotein (HDL; GM, *P* = 0.003, metabolome, *P* = 0.032, proteome, *P*=0.017, mRNA sequencing, *P*=0.011) and low-density lipoprotein (LDL; proteome, *P*=0.021).

### Differences in GM between the IGT and HC groups

**Figure 3:**
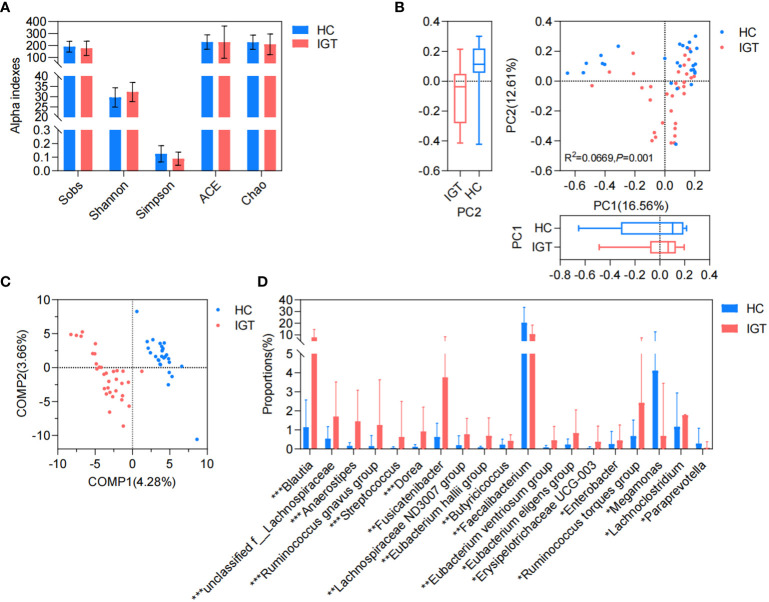
GM in IGT and HC groups. **(A)** Alpha indices represented the bacterial community richness, evenness and diversity. **(B)** Beta diversity based on Bray-Curtis’s distance in PCoA and Adonis analysis. **(C)** PLS-DA. **(D)** Differential genera both screened in the Wilcoxon rank-sum test and LEfSe analysis. HC, healthy control; IGT, impaired glucose tolerance; PC, principal component; COMP, component; GM, gut microbiota; PCoA, principal coordinate analysis; PLS-DA, partial least squares discriminant analysis; LEfSe, linear discriminant analysis effect size. *P<0.05, **P<0.01, ***P<0.001.

There was no significant difference observed in the richness, evenness, and diversity of the bacterial community between the IGT and HC groups as the similar values of alpha diversity indexes (Sobs, Shannon, Simpson, ACE, and Chao) (**Figure 3A**; **Supplementary Figure 3**). However, the PCoA and PLS-DA analysis revealed a distinct separation in the distribution between patients with IGT and HC volunteers based on the Bray-Curtis distance (**Figures 3B, C**). Moreover, the relative abundances of the genera in the IGT group altered significantly, and 21 genera were screened in the Wilcoxon rank-sum test, as well as 45 genera in LEfSe analysis (**Supplementary Tables 12, 13**), and there were 19 genera in common in both (**Figure 3D**), including some predominant genera with relative abundance > 1%, such as *Faecalibacterium*, *Fusicatenibacter*, *Blautia* and *Lachnoclostridium*. Significantly, *Blautia*, *Faecalibacterium* and *Dorea* displayed the same alterations in both T2D and IGT patients (**Figures 1C**, **3D**). In addition, ROC curve was constructed based on *Blautia*, *Fusicatenibacter* and *Butricococcus*, and the AUC was 0.97, which displayed credible diagnostic value (**Supplementary Figure 4**).

### Differential plasma metabolites between the IGT and HC groups

**Figure 4:**
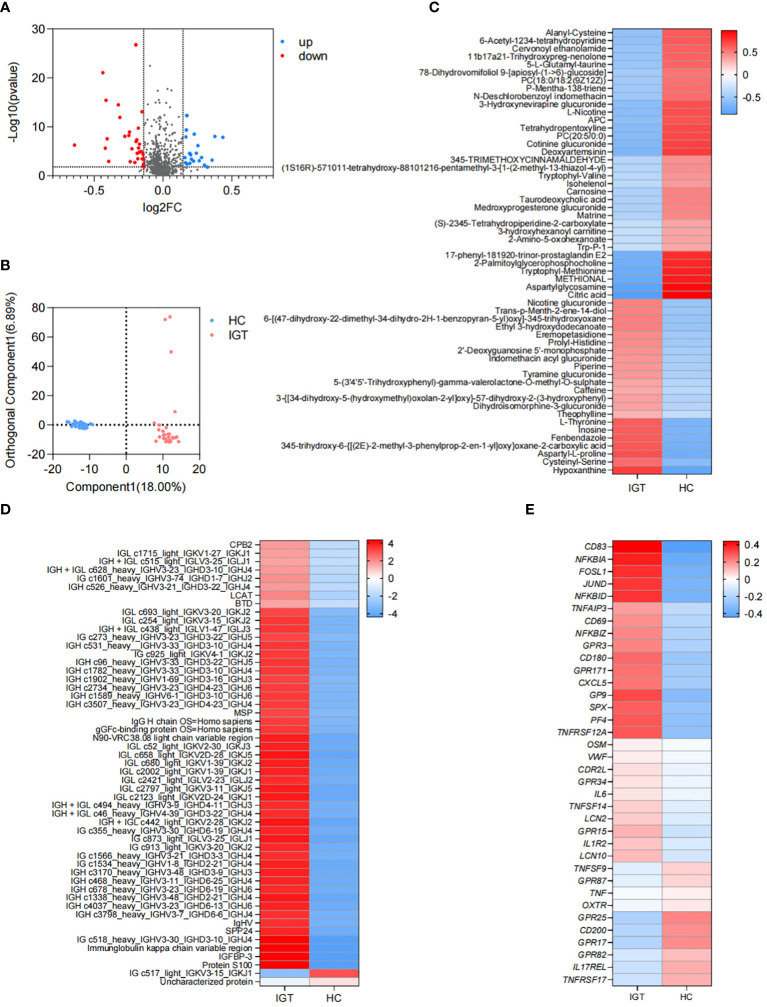
Differential plasma metabolites, proteins and DEGs in peripheral leukocytes in IGT and HC groups. **(A)** Volcano plots and **(B)** PLS-DA based on the plasma metabolite profiles in patients with IGT and HC volunteers. **(C)** Heatmap based on the differential plasma metabolites, **(D)** the representative differential plasma proteins and **(E)** the representative DEGs in peripheral leukocytes between IGT and HC groups. HC, healthy control; IGT, impaired glucose tolerance; FC, fold change; OPLS-DA, orthogonal partial least squares discriminant analysis; DEG, differential expressed gene.

There were 56 metabolites that showed different concentrations between IGT patients and HC volunteers. Among these metabolites, 22 significantly increased in the IGT group, while 34 decreased dramatically (**Figure 4A**; **Supplementary Table 14**). This resulted in a clear separation between the groups in the OPLS-DA (**Figure 4B**). Furthermore, this difference was even more pronounced in the heatmap (**Figure 4C**). Notably, the metabolite involved in secondary bile acid metabolism and derived from the GM - TUDCA, showed a decreasing trend in patients with IGT, and similar changes were observed in numerous metabolites involved in amino acid metabolism, including tryptophyl-methionine, tryptophyl-valine, and Trp-P-1, the latter being a carcinogenic heterocyclic amine.

### Differential plasma proteins between the IGT and HC groups

A total of 172 proteins exhibited significant differences between the IGT and HC groups. Twenty-one proteins showed decreased expression levels in the IGT group, while 151 proteins showed increased expression levels (**Supplementary Table 15**). The heatmap (**Figure 4D**) displayed some of these proteins, with a noteworthy observation that most of them were immunoglobulins. Additionally, certain proteins resembled blood coagulation factors and proteases, such as lysozyme C and carboxypeptidase. Interestingly, the insulin sensitizing hormone adiponectin D exhibited downregulation in the IGT group, while insulin-like growth factor II (IGF II) and IGFBP-3 were upregulated (**Supplementary Table 15**).

### DEGs in peripheral leukocytes between the IGT and HC groups

A total of 2561 DEGs were identified between IGT patients and HC volunteers. Among these DEGs, 1725 genes were upregulated in peripheral leukocytes in patients with IGT, while 836 genes were downregulated (**Supplementary Table 16**). **Figure 4E** displayed the immune- and endocrine-related DEGs. The heatmap revealed that these DEGs included clusters of differentiation (CDs) that regulate the differentiation and activation of leukocytes. Examples of downregulated CDs in the IGT group were CD80/180/302, while upregulated CDs were CD83/177. Additionally, numerous inflammatory genes, such as IL-6/10/11/12/13/17/27, tumor necrosis factors (TNFs), NFκB inhibitors, and matrix metallopeptidases (MMPs, MMP-9/23/28), were observed in patients with IGT (**Supplementary Table 16**). Notably, the gene encoding lipocalin 2 (LCN2) was also significantly upregulated in the IGT group (**Figure 4E**). Furthermore, the expression of pattern recognition receptors varied, with G protein-coupled receptors (GPR) 3/42/84/87 showing upregulation and GPR15/79/171 demonstrating downregulation (**Figure 4E**; **Supplementary Table 16**).

### Functional changes in KEGG pathways between the IGT and HC groups

**Figure 5:**
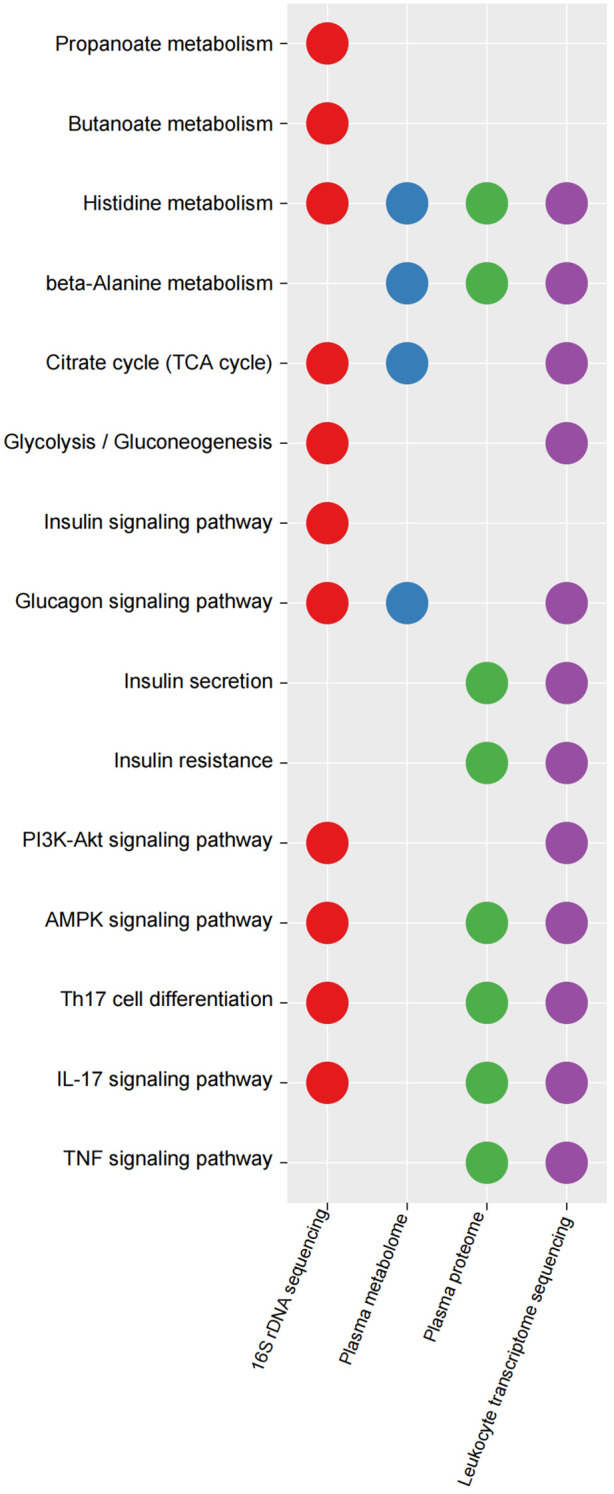
Key KEGG pathways mapped by differential KOs in GM, differential plasma metabolites/proteins and DEGs in peripheral leukocytes between IGT and HC groups. KEGG, Kyoto Encyclopedia of Genes and Genomes; KO, KEGG orthology; IGT, impaired glucose tolerance; DEG, differential expressed gene; HC, healthy control.

The 16S rDNA gene was annotated using TAX4FUN (version 0.3.1) and mapped to the KEGG database, resulting in the identification of 6022 KOs (**Supplementary Table 17**). To identify differential KOs between IGT and HC groups, we conducted Wilcoxon rank-sum tests and LEfSe analysis. In the former, 2682 KOs showed significant differences (*P* < 0.05, **Supplementary Table 18**), while the latter identified 408 key differential KOs shared in both analyses (LDA (linear discriminant analysis) > 2 and *P* < 0.05, **Supplementary Table 19**). Subsequently, we identified 144 KEGG pathways (**Supplementary Table 20**), several of which were related to glycometabolism, including the TCA cycle, glycolysis/gluconeogenesis, fructose and mannose metabolism, and galactose metabolism (**Supplementary Table 20**; **Figure 5**). Additionally, pathways associated with well-known bacterial metabolites, such as propanoate and butanoate, were also identified (**Supplementary Table 20**; **Figure 5**), and pathways involved in the regulation of blood glucose, such as insulin and glucagon signaling were also identified, as well as inflammatory pathways, including Th17 cell differentiation, IL-17 signaling pathway, AMPK signaling pathway, and PI3K-Akt signaling pathway (**Supplementary Table 20**; **Figure 5**). Interestingly, the KEGG pathways associated with differential metabolites/proteins in the plasma and the DEGs in peripheral leukocytes also exhibited involvement in endocrine and immune functions (**Figure 5**; **Supplementary Tables 21–23**). This finding suggests that these processes may play crucial roles in the progression of diabetes and its accompanying vasculopathy. Moreover, we found the pathway associated with histidine metabolism to be consistently present in all functional analyses. A metabolite in plasma linked to this pathway was identified as CARN (**Figure 5**; **Supplementary Table 21**).

### TUDCA and CARN derived from the GM regulated blood glucose and inflammation by inhibiting the expression of IGFBP-3 and IL-6

**Figure 6:**
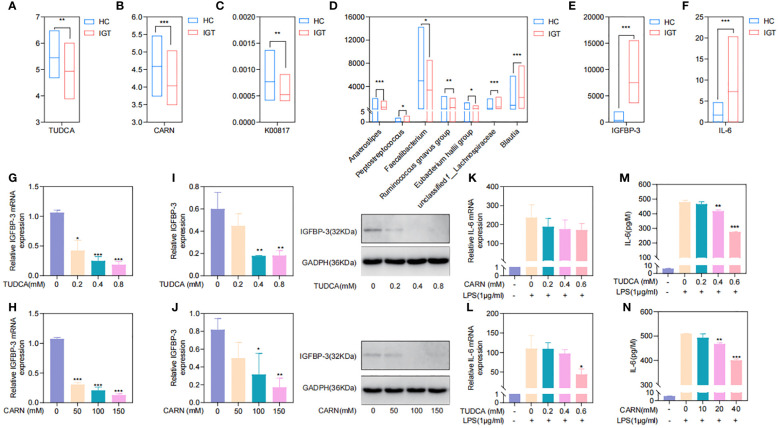
TUDCA and CARN derived from the GM regulated blood glucose and inflammation by inhibiting the expression of IGFBP-3 and IL-6. **(A)** TUDCA and **(B)** CARN decreased significantly in the plasma of IGT group. **(C)** The predicted KO based on the 16S rDNA gene related to the synthesis of CARN were downregulated in the IGT group. **(D)** Genera related to TUDCA and CARN based on the Spearman’s correlation coefficient. **(E)** IGFBP-3 and **(F)** IL-6 were both increasing in the IGT group. **(G-J)** TUDCA and CARN inhibited the expression of IGFBP-3 in the HepG2 cells. **(K-N)** TUDCA and CARN inhibited the expression of IL-6 induced by LPS in neutrophils from mice. HC, healthy control; IGT, impaired glucose tolerance; GM, gut microbiota; KO, KEGG orthology; TUDCA, taurodeoxycholic acid; CARN, carnsine; IGFBP-3, insulin-like growth factor binding protein-3; IL-6, interleukin-6; LPS, lipopolysaccharide. **(A–F)** *P<0.05, **P<0.01, ***P<0.001; **(G–J)** Comparing with blank control group (0 mM) *P<0.05, **P<0.01, ***P<0.001; **(K-N)** Comparing with group intervened with only LPS *P<0.05, **P<0.01, ***P<0.001.

**Figure 7:**
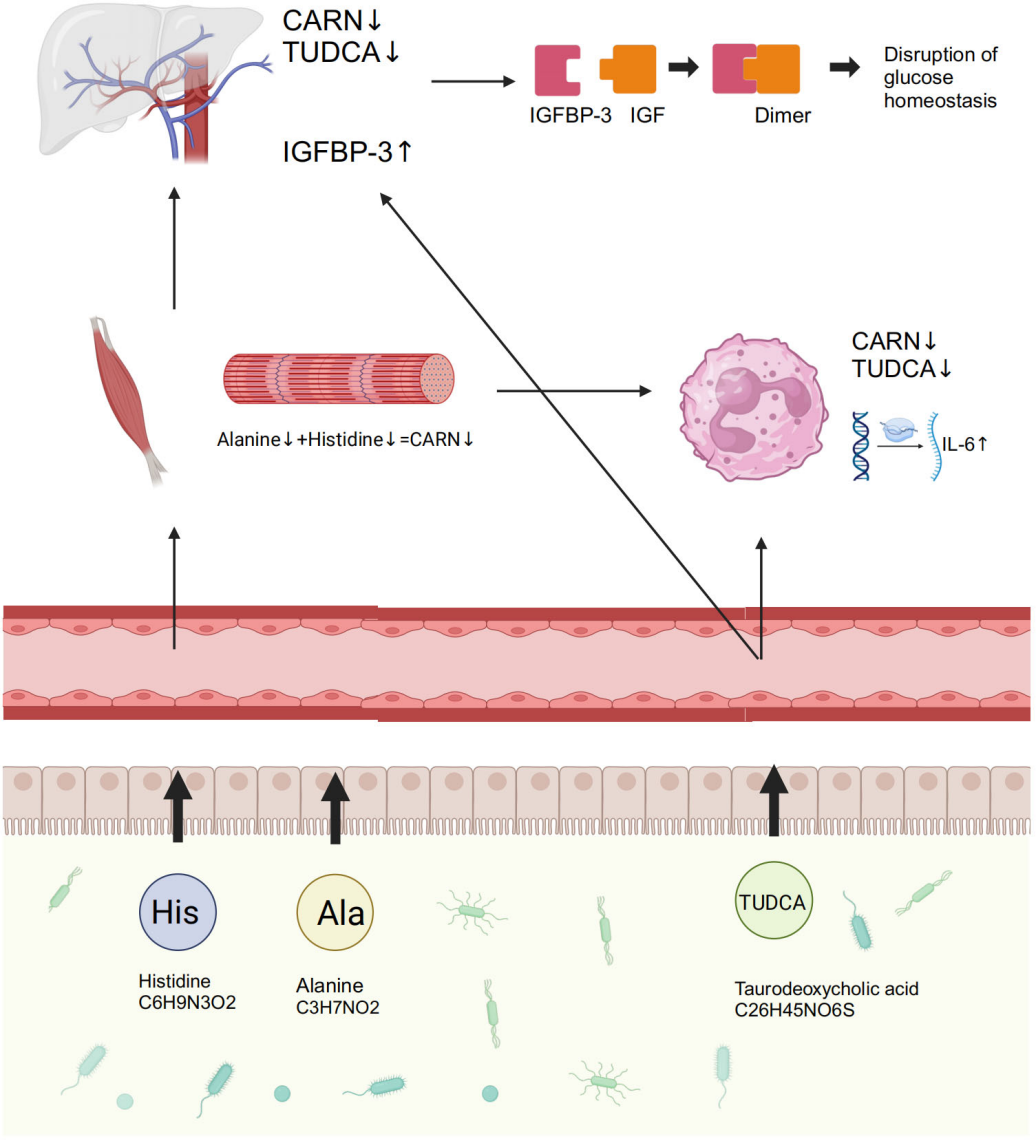
GM and its associated metabolites dysbiosis caused hyperglycemia and the related vasculopathy. GM, gut microbiota; TUDCA, taurodeoxycholic acid; CARN, carnosine; IGFBP-3, insulin-like growth factor binding protein-3; IL-6, interleukin-6; IGF, insulin-like growth factor.

To explore the regulatory mechanisms of GM on blood glucose and inflammation, we focused on the metabolites TUDCA and CARN, both of which were decreased in IGT (**Figures 6A, B**). It is well established that TUDCA production is closely related to GM. However, there is currently no research reporting the influence of GM on the biosynthesis of CARN. Interestingly, among the predicted KOs based on the 16S rDNA genes, K00817 (histidinol-phosphate aminotransferase) - the gene that monitors CARN production - showed a reduction in expression in the IGT group (**Figure 6C**). Previous studies have demonstrated the ability of the GM to synthesize alanine and histidine. However, it is important to note that the synthesis of these amino acids serves as the rate-limiting step in myostatin synthesis. This suggests that GM can potentially supply precursor species for the synthesis of CARN. Analysis of Spearman’s correlation coefficients revealed that only *Faecalibacterium* (R=0.82, *P*=0.04; R=0.71, *P*=0.03) exhibited a positive influence on the production of both TUDCA and CARN (**Figure 6D**; **Supplementary Figure 5**). In contrast, *Anaerostipes* (R=-0.56, *P*=0.04; R=-0.61, *P*=0.02), *unclassified f_Lachnospiraceae* (R=-0.48, *P*=0.03; R=-0.71, *P*=0.01), *Ruminococcus gnavus group* (R=-0.48, *P*=0.02; R=-0.68, *P*=0.04), *Eubacterium hallii group* (R=-0.55, *P*=0.02; R=-0.66, *P*=0.04), *Peptostreptococcus* (R=-0.56, *P*=0.03; R=-0.81, *P*=0.02), and *Blautia* (R=-0.72, *P*=0.01; R=-0.48, *P*=0.02) all displayed negative correlations with the concentrations of these two metabolite (**Figure 6D**; **Supplementary Figure 5**), and *Blautia* was also considered to be one of the potential biomarkers in the ROC curve (**Supplementary Figure 4**). As mentioned in previous research, TUDCA has been identified as a regulator of inflammation progression, while CARN has the ability to impact blood glucose levels by monitoring the expression of IGFBP (Wang et al., 2018; Kusaczuk, 2019). Fortunately, we identified IGFBP-3 among the differential proteins in plasma and IL-6 among DEGs obtained from mRNA sequencing of leukocytes as targets influenced by TUDCA and CARN. Consistent with our expectations, the levels of both IGFBP-3 and IL-6 increased significantly in patients with IGT (**Figures 6E, F**). Furthermore, in the subsequent cell experiments, TUDCA and CARN exhibited remarkable inhibitory effects on IGFBP-3 and IL-6, respectively (**Figures 6G–N**). In summary, patients with IGT demonstrated decreased levels of TUDCA and CARN, which originate from GM dysbiosis. This decrease led to a reduced suppression on the expression of IGFBP-3 and IL-6. Overexpression of these proteins may play significant roles in insulin resistance and the development of vasculopathy during the onset of diabetes (Rahman et al., 2007; Cleland et al., 2013; Alwin Robert and Al Dawish, 2019; Malone and Hansen, 2019) (**Figure 7**).

## Discussion

This study investigated the GM metagenome in patients with T2D, and the results revealed that variations in cholate and histidine metabolism, which are associated with changes in GM, played significant roles in diabetes. The prominent genera identified included *Bacteroides, Prevotella, Faecalibacterium, Bifidobacterium, Ruminococcus, Blautia*, and *Lachnoclostridium*. This is highly similar to previous studies (Gurung et al., 2020; Yang et al., 2021). Subsequently, to investigate the specific mechanisms by which the GM and its metabolites contribute to the development of diabetes and its complications, we recruited individuals with IGT, representing the prediabetic state, along with healthy volunteers. Testing included 16S rDNA sequencing, mRNA sequencing of peripheral leukocytes, plasma metabolome, and proteome. Encouragingly, we discovered that TUDCA, a component of cholate metabolism, and CARN, a downstream metabolite of histidine, both originating from the GM, played crucial roles in regulating blood glucose levels and vascular inflammation. The increased abundance of *Blautia* in GM, coupled with the decreased abundance of *Faecalibacterium*, leads to reduced synthesis of TUDCA and CARN in the plasma. Previous studies have also found abnormal expression of TUDCA in patients with diabetes, as well as the precursors of CARN -histine and alanine - in diabetic mice (Chen et al., 2019; Guasch-Ferre et al., 2020; Liu et al., 2021). Consequently, the significant reduction in the expression of IGFBP-3 and IL-6, as a result of the inhibitory effects of these metabolites, leads to the emergence of abnormal blood glucose levels and the accompanying vasculopathy during the occurrence of diabetes in this study.

Undoubtedly, the GM possesses the capacity to ferment food components into metabolites that can be absorbed by the body. These metabolites, in turn, play pivotal roles in regulating nutrient intake, energy metabolism, and immune homeostasis within the organism (Len et al., 2004; Medini et al., 2005; Bhardwaj and Somvanshi, 2017; Zhang et al., 2017). The dysbiosis of the GM in patients with diabetes has been extensively investigated (Sato et al., 2014; Fernandes et al., 2019). The most extensively studied mechanism involves the reduction in butyrate-producing bacteria, which leads to a consequent decrease in the concentration of butyrate (Noureldein et al., 2020; Arora and Tremaroli, 2021). However, *Faecalibacterium*, the representative butyrate-producing bacteria (Zhou et al., 2018), was also found in our study - both in T2D and IGT research, which indicated the significance of short-chain fatty acid metabolism in diabetes occurrence. Unfortunately, we did not identify short-chain fatty acids as differential metabolites between the T2D/IGT and HC groups in our study. However, throughout the entire research process, from IGT to T2D, we consistently focused on studying the metabolism of cholate and L-histidine. Previous studies have shown that alterations in cholate impact fat digestion and glucose metabolism, enhance insulin sensitivity and reduce hepatic glucose production through the farnesoid X receptor (Watanabe et al., 2006). The pathway related to L-histidine biosynthesis plays a critical role in regulating inflammation and oxidative stress, while both these two were key elements in the pathogenesis of T2D (Miyazaki et al., 2019). Notably, L-histidine can be converted into histamine and CARN, the substances that potentially benefit insulin sensitivity and anti-inflammatory responses (Hirasawa, 2019). Therefore, these two metabolic pathways not only play central roles in the progression of T2D, but also may serve as potential targets for the future treatments.

*Blautia*, a widely present probiotic bacteria and dominant genus, is commonly found in the feces and intestines of mammals (Rodriguez et al., 2020). Numerous studies have confirmed its association with obesity, diabetes, cancer, and various inflammatory diseases (Houtkooper et al., 2010; Hwang and Song, 2017; Wang et al., 2017; Kirkland and Meyer-Ficca, 2018). It is known that *Blautia* influences the conversion of bile acids into secondary bile acids, for example, TUDCA, by regulating the related enzymes, such as 7α-dehydrogenase, thereby modulates the enterohepatic circulation and the insulin sensitivity (Nakamura et al., 2016). Moreover, *Blautia* indirectly affects the synthesis of CARN by influencing the availability of L-carnitine through its impact on the intestinal pH and microenvironment (Wang et al., 2023). *Faecalibacterium*, as a primary butyrate-producing bacterium, promotes the bile acid metabolism as result of its anti-inflammatory effect and maintains intestinal barrier integrity, thus to stabilize the concentration of TUDCA (Miquel et al., 2013). And it also enhances the intestinal environment to increase the availability of L-carnitine and promote the synthesis of CARN (Hales, 2019). However, the role of *Blautia* in the biosynthesis of CARN remains understudied, and there is limited research investigating the correlation between *Faecalibacterium* and TUDCA and CARN. Our study results uncover the significant impact of the balance between *Blautia* and *Faecalibacterium* on the production of TUDCA and CARN. It is possible that variations in the abundances of coding genes in the bacterial genome, which are associated with these genera, play key roles in their biosynthetic alterations. Nevertheless, further exploration is needed to elucidate the specific mechanism.

TUDCA, a conjugated natural secondary bile acid, is widely present in humans and animals. It serves to protect hepatocytes, promote the transport and secretion of bile acids, and has been found to lower blood glucose levels by enhancing insulin sensitivity through its role as a molecular chaperone (Kars et al., 2010; Bronczek et al., 2019; da Silva et al., 2021). TUDCA enhances the viability and migration ability of Schwann cells under high glucose conditions, suggesting its potential therapeutic effects on diabetic peripheral neuropathy (Wang et al., 2024). Additionally, TUDCA inhibits the endoplasmic reticulum stress response, therefore mitigates insulin resistance (Kars et al., 2010; Vettorazzi et al., 2017), as well as protects cells from inflammatory damage and lowers the systematic inflammation (Alhasani et al., 2020), while the inflammation indicated as elevated CRP, TNF-α, and IL-6 also can drive the dysfunction of pancreatic β-cells and increase the insulin resistance in patients with T2D (Donath and Shoelson, 2011). CARN is an endogenous dipeptide with diverse biological functions *in vivo (* Lievens et al., 2024). These include anti-inflammation, antioxidation, anti-glycosylation, metal ion chelation, and promotion of wound healing (Alhamdani et al., 2007; Jukic et al., 2021; Schwank-Xu et al., 2021). Previous studies have revealed the potential of CARN in regulating blood glucose levels, as it protects the cells responsible for controlling blood glucose, enhances their sensitivity to glucose, and promotes insulin secretion (Boldyrev and AldiniW Derave, 2013; Liu et al., 2020), while CARN supplementation can reduce fasting blood glucose, serum triglyceride levels, advanced glycation end products, and TNF-α in T2D patients (Houjeghani et al., 2018), and inhibit the production of extracellular matrix components and transforming growth factor-β in a high glucose environment to protect against diabetic nephropathy (Janssen et al., 2005). Meanwhile, CARN restricts the migration and activation of the inflammatory cells, decreases the production of pro-inflammatory cytokines (such as TNF-α and IL-6) and the reactive oxygen species (Abdin et al., 2010), while peroxidation can damage the vascular endothelial cell and exacerbate the progression of the diabetic complications (Da Ros et al., 2004). The decrease in concentrations of TUDCA and CARN among the patients with IGT in our study aligns with their recognized roles in blood glucose regulation. This highlights the potential of these two metabolites as medication to potentially halt or reverse the progression from IGT to diabetes.

Furthermore, we demonstrated the inhibitory effect of TUDCA and CARN on IGFBP-3 in HepG2 cells. IGFBP-3, the most abundant IGF binding protein in the blood, plays a crucial role in transporting IGFs by safeguarding them from degradation and modifying their interaction with specific receptors through structural changes (Ranke, 2015; Varma Shrivastav et al., 2020). Moreover, IGFBP-3 is implicated in insulin resistance (Chan et al., 2005; Mohanraj et al., 2013). Hence, the decrease in TUDCA and CARN resulting from GM dysbiosis in patients with IGT leads to elevated blood glucose levels. This reduction in TUDCA and CARN diminishes the inhibitory effect on IGFBP-3, thereby promoting subsequent insulin resistance. In addition, we also directed our attention to the significance of inflammation caused by GM dysbiosis, which has the potential to contribute to vasculopathy in the presence of hyperglycemia (Calle and Fernandez, 2012). It has been indicated that inflammation serves as a significant pathophysiological basis for various vascular diseases, notably atherosclerosis and hypertension (Whiteford et al., 2016). The increased levels of inflammatory cytokines not only damage vascular endothelial cells but also lead to elevated oxidative stress, ultimately contributing to the development of fundamental vascular injury throughout disease progression (Steven et al., 2019). Regrettably, no significant difference was observed in the concentration of IL-17 between the IGT and HC groups (**Supplementary Figure 6**), despite its confirmed importance in diabetes incidence (Xiao et al., 2017; Huang et al., 2020). Notably, pathways related to IL-17 were identified in our functional analyses (**Figure 5**). Consequently, we investigated the impact of TUDCA and CARN on the expression of IL-6, an extensively recognized cytokine involved in inflammation that can activate macrophages and modulate the differentiation of B/T cells and thymocytes (Ma et al., 2012; Dixit et al., 2018). Additionally, IL-6 plays a critical role in various cellular processes, including the maturation of megakaryocytes into platelets, the activation of hematopoietic stem cells, and the differentiation and proliferation of multiple cell types, such as osteoblasts, keratinocytes, glomerular membrane cells, myeloma, and plasmacytoma cells (Akbari and Hassan-Zadeh, 2018; Robinson et al., 2020). Our study revealed that the reduction in TUDCA and CARN in the IGT group led to an increase in the expression of IL-6. This inflammatory response in the blood may contribute to the development of vasculopathy, which could serve as the underlying pathology for diabetic complications.

However, due to the small sample size and a focus on the specific population, the results of this study are restricted, and we plan to employ a large-scale study with long-term follow-up in the future to better understand the long-term effects of these genera and their metabolites during the progression of T2D. Additionally, investigating these genera and their metabolites in diverse populations will enhance the global understanding of T2D and promote to develop new targeted therapies (Domingueti et al., 2016; Jha et al., 2018).

## Conclusions

GM dysbiosis, characterized by increased *Blautia* and decreased *Faecalibacterium*, occurs throughout the progression from IGT to diabetes. This dysbiosis leads to reduced levels of TUDCA and CARN, which in turn alleviate their inhibition on the synthesis of IGFBP-3 and the expression of IL-6, ultimately resulting in hyperglycemia and related vasculopathy. This finding advances our understanding of the pathogenesis of IGT and diabetes, provides the potential for early disease screening and clinical diagnosis, and suggests possible therapeutic targets for preventing the progression from IGT to diabetes.

## Data Availability

The datasets presented in this study can be found in online repositories. The names of the repository/repositories and accession number(s) can be found in the **Supplementary Material** (**Supplementary Table 24**).
