# Supplementary material for: The gut microbiota intervenes in glucose tolerance and inflammation by regulating the biosynthesis of taurodeoxycholic acid and carnosine
Source: Front Cell Infect Microbiol. 2024 Aug 14;14:1423662. doi: 10.3389/fcimb.2024.1423662 (PMC11351283; doi:10.3389/fcimb.2024.1423662)
Supplement: Supplementary file 2 [file DataSheet1.pdf]

BD FACSDiva 8.0.1

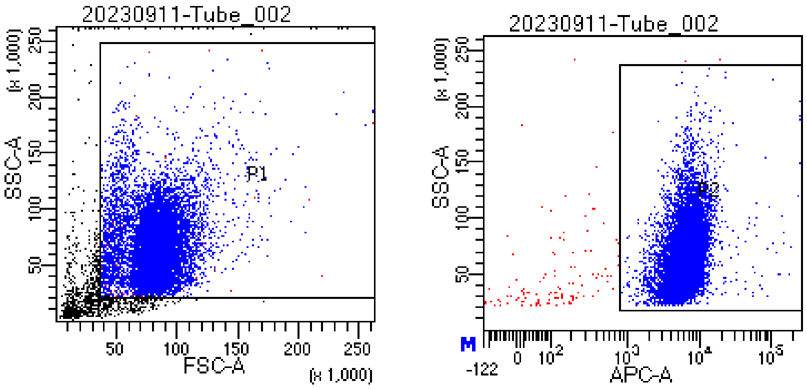

Tube: Tube_002

Population	#Events	%Parent	%Total
All Events	11,058	####	100.0
P1	10,000	90.4	90.4
P2	9,880	98.8	89.3

Experiment Name:	NE
Specimen Name:	20230911
Tube Name:	Tube_002
Record Date:	Sep 11, 2023 7:00:30 PM
$OP:	Administrator
GUID:	30479061-6745-49c5-953c-f13f...

Population	#Events	%Parent	FSC-A Mean	SSC-A Mean
All Events	11,058	####	76,613	65,489
P1	10,000	90.4	81,147	68,781
P2	9,880	98.8	81,209	68,986

Supplementary Figure 1 Fluorescence activated cell sorting results of neutrophils.

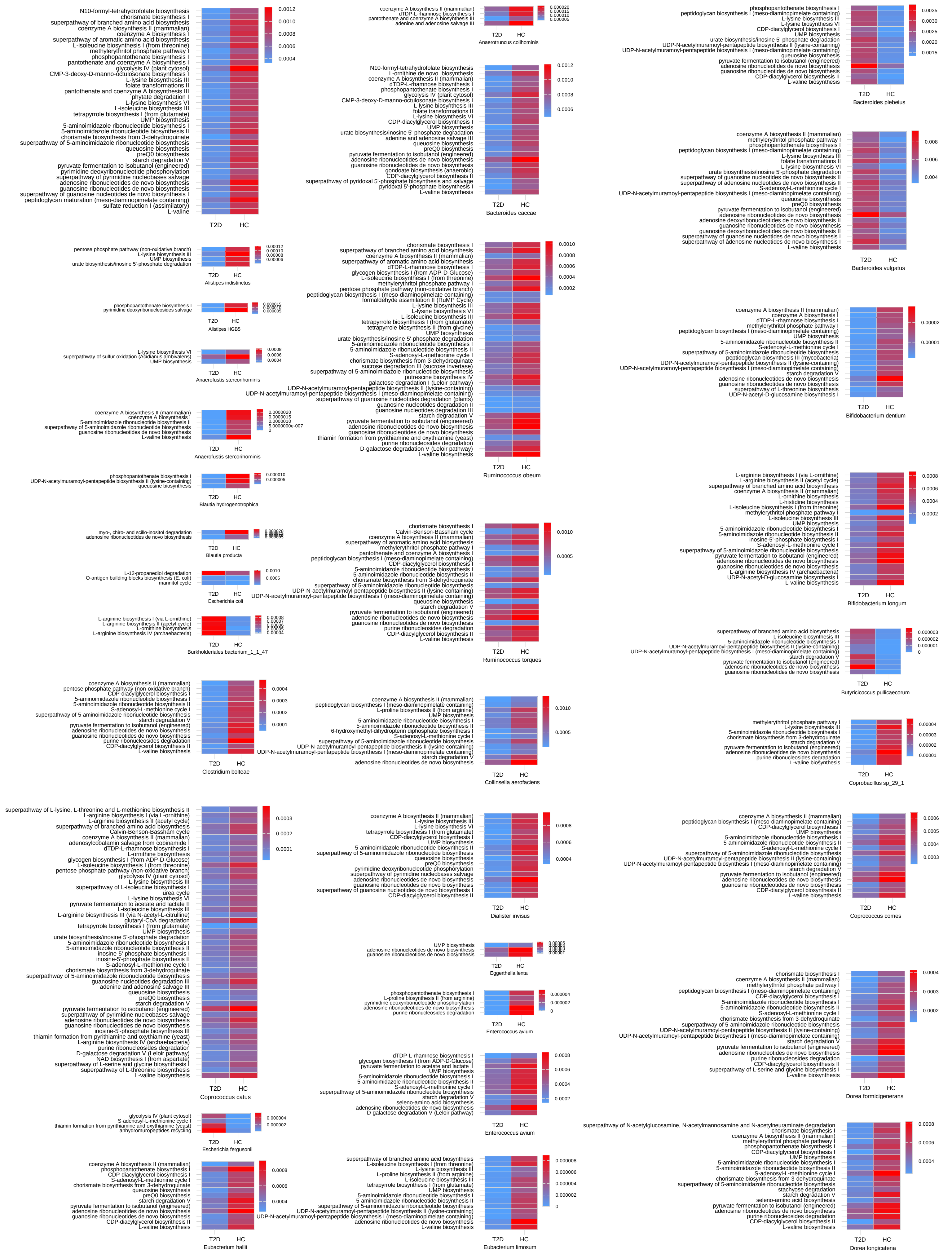
Anaerotruncus colihominis
Bacteroides plebeius
Bacteroides caccae
Bacteroides vulgatus
Alistipes indistinctus
Alistipes HGB5
Anaerofustis stercorihominis
Anaerofustis stercorihominis
Ruminococcus obeum
Bifidobacterium dentium
Blautia hydrogenotrophica
Blautia producta
Escherichia coli
Burkholderiales bacterium_1_1_47
Ruminococcus torques
Bifidobacterium longum
Butyricicoccus pullicaecorum
Clostridium bolteae
Collinsella aerofaciens
Coprobacillus sp_29_1
Coprococcus catus
Dialister invisus
Coprococcus comes
Eggerthella lenta
Enterococcus avium
Enterococcus avium
Dorea formicigenerans
Escherichia fergusonii
Eubacterium hallii
Eubacterium limosum
Dorea longicatena

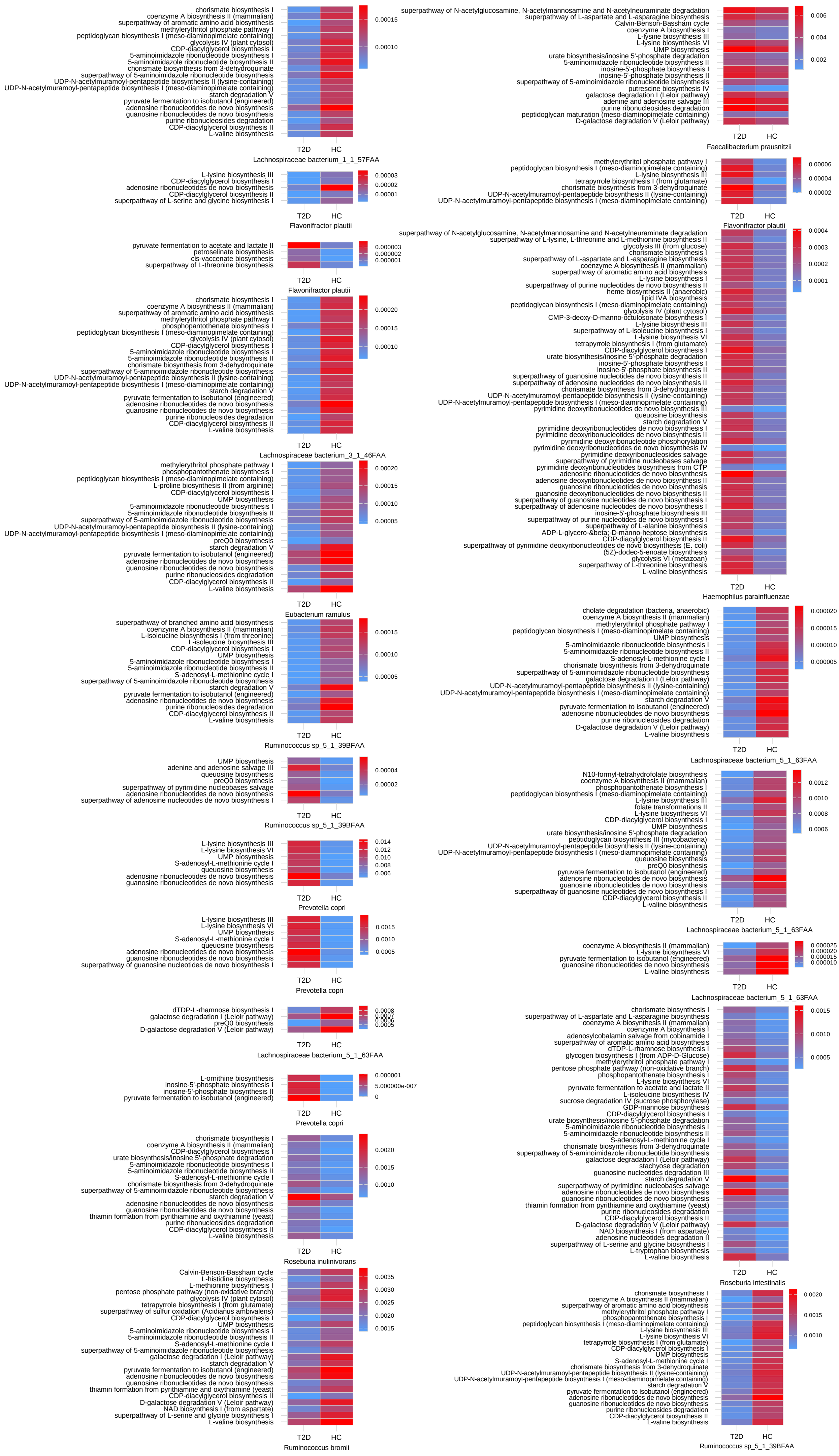
chorismate biosynthesis I
coenzyme A biosynthesis II (mammalian)
superpathway of aromatic amino acid biosynthesis
methylerythritol phosphate pathway I
peptidoglycan biosynthesis I (meso-diaminopimelate containing)
glycolysis IV (plant cytosol)
CDP-diacylglycerol biosynthesis I
5-aminoimidazole ribonucleotide biosynthesis I
5-aminoimidazole ribonucleotide biosynthesis II
chorismate biosynthesis from 3-dehydroquinate
superpathway of 5-aminoimidazole ribonucleotide biosynthesis
UDP-N-acetylmuramoyl-pentapeptide biosynthesis II (lysine-containing)
UDP-N-acetylmuramoyl-pentapeptide biosynthesis I (meso-diaminopimelate containing)
starch degradation V
pyruvate fermentation to isobutanol (engineered)
adenosine ribonucleotides de novo biosynthesis
guanosine ribonucleotides de novo biosynthesis
purine ribonucleosides degradation
CDP-diacylglycerol biosynthesis II
L-valine biosynthesis
0.00015
0.00010
T2D
HC
Lachnospiraceae bacterium_1_1_57FAA
L-lysine biosynthesis III
CDP-diacylglycerol biosynthesis I
adenosine ribonucleotides de novo biosynthesis
CDP-diacylglycerol biosynthesis II
superpathway of L-serine and glycine biosynthesis I
0.00003
0.00002
0.00001
T2D
HC
Flavonifractor plautii
pyruvate fermentation to acetate and lactate II
petroselinate biosynthesis
cis-vaccenate biosynthesis
superpathway of L-threonine biosynthesis
0.000003
0.000002
0.000001
T2D
HC
Flavonifractor plautii
chorismate biosynthesis I
coenzyme A biosynthesis II (mammalian)
superpathway of aromatic amino acid biosynthesis
methylerythritol phosphate pathway I
phosphopantothenate biosynthesis I
peptidoglycan biosynthesis I (meso-diaminopimelate containing)
glycolysis IV (plant cytosol)
CDP-diacylglycerol biosynthesis I
5-aminoimidazole ribonucleotide biosynthesis I
5-aminoimidazole ribonucleotide biosynthesis II
chorismate biosynthesis from 3-dehydroquinate
superpathway of 5-aminoimidazole ribonucleotide biosynthesis
UDP-N-acetylmuramoyl-pentapeptide biosynthesis II (lysine-containing)
UDP-N-acetylmuramoyl-pentapeptide biosynthesis I (meso-diaminopimelate containing)
starch degradation V
pyruvate fermentation to isobutanol (engineered)
adenosine ribonucleotides de novo biosynthesis
guanosine ribonucleotides de novo biosynthesis
purine ribonucleosides degradation
CDP-diacylglycerol biosynthesis II
L-valine biosynthesis
0.00020
0.00015
0.00010
T2D
HC
Lachnospiraceae bacterium_3_1_46FAA
methylerythritol phosphate pathway I
phosphopantothenate biosynthesis I
peptidoglycan biosynthesis I (meso-diaminopimelate containing)
L-proline biosynthesis II (from arginine)
CDP-diacylglycerol biosynthesis I
UMP biosynthesis
5-aminoimidazole ribonucleotide biosynthesis I
5-aminoimidazole ribonucleotide biosynthesis II
superpathway of 5-aminoimidazole ribonucleotide biosynthesis
UDP-N-acetylmuramoyl-pentapeptide biosynthesis II (lysine-containing)
UDP-N-acetylmuramoyl-pentapeptide biosynthesis I (meso-diaminopimelate containing)
preQ0 biosynthesis
starch degradation V
pyruvate fermentation to isobutanol (engineered)
adenosine ribonucleotides de novo biosynthesis
guanosine ribonucleotides de novo biosynthesis
purine ribonucleosides degradation
CDP-diacylglycerol biosynthesis II
L-valine biosynthesis
0.00020
0.00015
0.00010
0.00005
T2D
HC
Eubacterium ramulus
superpathway of branched amino acid biosynthesis
coenzyme A biosynthesis II (mammalian)
L-isoleucine biosynthesis I (from threonine)
L-isoleucine biosynthesis III
CDP-diacylglycerol biosynthesis I
UMP biosynthesis
5-aminoimidazole ribonucleotide biosynthesis I
5-aminoimidazole ribonucleotide biosynthesis II
S-adenosyl-L-methionine cycle I
superpathway of 5-aminoimidazole ribonucleotide biosynthesis
starch degradation V
pyruvate fermentation to isobutanol (engineered)
adenosine ribonucleotides de novo biosynthesis
purine ribonucleosides degradation
CDP-diacylglycerol biosynthesis II
L-valine biosynthesis
0.00015
0.00010
0.00005
T2D
HC
Ruminococcus sp_5_1_39BFAA
UMP biosynthesis
adenine and adenosine salvage III
queuosine biosynthesis
preQ0 biosynthesis
superpathway of pyrimidine nucleobases salvage
adenosine ribonucleotides de novo biosynthesis
superpathway of adenosine nucleotides de novo biosynthesis I
0.00004
0.00002
T2D
HC
Ruminococcus sp_5_1_39BFAA
L-lysine biosynthesis III
L-lysine biosynthesis VI
UMP biosynthesis
S-adenosyl-L-methionine cycle I
queuosine biosynthesis
adenosine ribonucleotides de novo biosynthesis
guanosine ribonucleotides de novo biosynthesis
0.014
0.012
0.010
0.008
0.006
T2D
HC
Prevotella copri
L-lysine biosynthesis III
L-lysine biosynthesis VI
UMP biosynthesis
S-adenosyl-L-methionine cycle I
queuosine biosynthesis
adenosine ribonucleotides de novo biosynthesis
guanosine ribonucleotides de novo biosynthesis
superpathway of guanosine nucleotides de novo biosynthesis I
0.0015
0.0010
0.0005
T2D
HC
Prevotella copri
dTDP-L-rhamnose biosynthesis I
galactose degradation I (Leloir pathway)
preQ0 biosynthesis
D-galactose degradation V (Leloir pathway)
0.0008
0.0007
0.0006
0.0005
T2D
HC
Lachnospiraceae bacterium_5_1_63FAA
L-ornithine biosynthesis
inosine-5'-phosphate biosynthesis I
inosine-5'-phosphate biosynthesis II
pyruvate fermentation to isobutanol (engineered)
0.000001
5.000000e-007
0
T2D
HC
Prevotella copri
chorismate biosynthesis I
coenzyme A biosynthesis II (mammalian)
CDP-diacylglycerol biosynthesis I
urate biosynthesis/inosine 5'-phosphate degradation
5-aminoimidazole ribonucleotide biosynthesis I
5-aminoimidazole ribonucleotide biosynthesis II
S-adenosyl-L-methionine cycle I
chorismate biosynthesis from 3-dehydroquinate
superpathway of 5-aminoimidazole ribonucleotide biosynthesis
starch degradation V
adenosine ribonucleotides de novo biosynthesis
guanosine ribonucleotides de novo biosynthesis
thiamin formation from pyrithiamine and oxythiamine (yeast)
purine ribonucleosides degradation
CDP-diacylglycerol biosynthesis II
L-valine biosynthesis
0.0020
0.0015
0.0010
T2D
HC
Roseburia inulinivorans
Calvin-Benson-Bassham cycle
L-histidine biosynthesis
L-methionine biosynthesis I
pentose phosphate pathway (non-oxidative branch)
glycolysis IV (plant cytosol)
tetrapyrrole biosynthesis I (from glutamate)
superpathway of sulfur oxidation (Acidianus ambivalens)
CDP-diacylglycerol biosynthesis I
UMP biosynthesis
5-aminoimidazole ribonucleotide biosynthesis I
5-aminoimidazole ribonucleotide biosynthesis II
S-adenosyl-L-methionine cycle I
superpathway of 5-aminoimidazole ribonucleotide biosynthesis
galactose degradation I (Leloir pathway)
starch degradation V
pyruvate fermentation to isobutanol (engineered)
adenosine ribonucleotides de novo biosynthesis
guanosine ribonucleotides de novo biosynthesis
thiamin formation from pyrithiamine and oxythiamine (yeast)
CDP-diacylglycerol biosynthesis II
D-galactose degradation V (Leloir pathway)
NAD biosynthesis I (from aspartate)
superpathway of L-serine and glycine biosynthesis I
L-valine biosynthesis
0.0035
0.0030
0.0025
0.0020
0.0015
T2D
HC
Ruminococcus bromii
superpathway of N-acetylglucosamine, N-acetylmannosamine and N-acetylneuraminate degradation
superpathway of L-aspartate and L-asparagine biosynthesis
Calvin-Benson-Bassham cycle
coenzyme A biosynthesis I
L-lysine biosynthesis III
L-lysine biosynthesis VI
UMP biosynthesis
urate biosynthesis/inosine 5'-phosphate degradation
5-aminoimidazole ribonucleotide biosynthesis II
inosine-5'-phosphate biosynthesis I
inosine-5'-phosphate biosynthesis II
superpathway of 5-aminoimidazole ribonucleotide biosynthesis
putrescine biosynthesis IV
galactose degradation I (Leloir pathway)
adenine and adenosine salvage III
purine ribonucleosides degradation
peptidoglycan maturation (meso-diaminopimelate containing)
D-galactose degradation V (Leloir pathway)
0.006
0.004
0.002
T2D
HC
Faecalibacterium prausnitzii
methylerythritol phosphate pathway I
peptidoglycan biosynthesis I (meso-diaminopimelate containing)
L-lysine biosynthesis III
tetrapyrrole biosynthesis I (from glutamate)
chorismate biosynthesis from 3-dehydroquinate
UDP-N-acetylmuramoyl-pentapeptide biosynthesis II (lysine-containing)
UDP-N-acetylmuramoyl-pentapeptide biosynthesis I (meso-diaminopimelate containing)
0.00006
0.00004
0.00002
T2D
HC
Flavonifractor plautii
superpathway of N-acetylglucosamine, N-acetylmannosamine and N-acetylneuraminate degradation
superpathway of L-lysine, L-threonine and L-methionine biosynthesis II
glycolysis III (from glucose)
chorismate biosynthesis I
superpathway of L-aspartate and L-asparagine biosynthesis
coenzyme A biosynthesis II (mammalian)
superpathway of aromatic amino acid biosynthesis
L-lysine biosynthesis I
superpathway of purine nucleotides de novo biosynthesis II
heme biosynthesis II (anaerobic)
lipid IVA biosynthesis
peptidoglycan biosynthesis I (meso-diaminopimelate containing)
glycolysis IV (plant cytosol)
CMP-3-deoxy-D-manno-octulosonate biosynthesis I
L-lysine biosynthesis III
superpathway of L-isoleucine biosynthesis I
L-lysine biosynthesis VI
tetrapyrrole biosynthesis I (from glutamate)
CDP-diacylglycerol biosynthesis I
urate biosynthesis/inosine 5'-phosphate degradation
inosine-5'-phosphate biosynthesis I
inosine-5'-phosphate biosynthesis II
superpathway of guanosine nucleotides de novo biosynthesis II
superpathway of adenosine nucleotides de novo biosynthesis II
chorismate biosynthesis from 3-dehydroquinate
UDP-N-acetylmuramoyl-pentapeptide biosynthesis II (lysine-containing)
UDP-N-acetylmuramoyl-pentapeptide biosynthesis I (meso-diaminopimelate containing)
pyrimidine deoxyribonucleotides de novo biosynthesis III
queuosine biosynthesis
starch degradation V
pyrimidine deoxyribonucleotides de novo biosynthesis I
pyrimidine deoxyribonucleotides de novo biosynthesis II
pyrimidine deoxyribonucleotide phosphorylation
pyrimidine deoxyribonucleotides de novo biosynthesis IV
pyrimidine deoxyribonucleosides salvage
superpathway of pyrimidine nucleobases salvage
pyrimidine deoxyribonucleotides biosynthesis from CTP
adenosine ribonucleotides de novo biosynthesis
adenosine deoxyribonucleotides de novo biosynthesis II
guanosine ribonucleotides de novo biosynthesis
guanosine deoxyribonucleotides de novo biosynthesis II
superpathway of guanosine nucleotides de novo biosynthesis I
superpathway of adenosine nucleotides de novo biosynthesis I
inosine-5'-phosphate biosynthesis III
superpathway of purine nucleotides de novo biosynthesis I
superpathway of L-alanine biosynthesis
ADP-L-glycero-β-D-manno-heptose biosynthesis
CDP-diacylglycerol biosynthesis II
superpathway of pyrimidine deoxyribonucleotides de novo biosynthesis (E. coli)
(5Z)-dodec-5-enoate biosynthesis
glycolysis VI (metazoan)
superpathway of L-threonine biosynthesis
L-valine biosynthesis
0.0004
0.0003
0.0002
0.0001
T2D
HC
Haemophilus parainfluenzae
cholate degradation (bacteria, anaerobic)
coenzyme A biosynthesis II (mammalian)
methylerythritol phosphate pathway I
peptidoglycan biosynthesis I (meso-diaminopimelate containing)
UMP biosynthesis
5-aminoimidazole ribonucleotide biosynthesis I
5-aminoimidazole ribonucleotide biosynthesis II
S-adenosyl-L-methionine cycle I
chorismate biosynthesis from 3-dehydroquinate
superpathway of 5-aminoimidazole ribonucleotide biosynthesis
galactose degradation I (Leloir pathway)
UDP-N-acetylmuramoyl-pentapeptide biosynthesis II (lysine-containing)
UDP-N-acetylmuramoyl-pentapeptide biosynthesis I (meso-diaminopimelate containing)
starch degradation V
pyruvate fermentation to isobutanol (engineered)
adenosine ribonucleotides de novo biosynthesis
purine ribonucleosides degradation
D-galactose degradation V (Leloir pathway)
L-valine biosynthesis
0.000020
0.000015
0.000010
0.000005
T2D
HC
Lachnospiraceae bacterium_5_1_63FAA
N10-formyl-tetrahydrofolate biosynthesis
coenzyme A biosynthesis II (mammalian)
phosphopantothenate biosynthesis I
peptidoglycan biosynthesis I (meso-diaminopimelate containing)
L-lysine biosynthesis III
folate transformations II
L-lysine biosynthesis VI
CDP-diacylglycerol biosynthesis I
UMP biosynthesis
urate biosynthesis/inosine 5'-phosphate degradation
peptidoglycan biosynthesis III (mycobacteria)
UDP-N-acetylmuramoyl-pentapeptide biosynthesis II (lysine-containing)
UDP-N-acetylmuramoyl-pentapeptide biosynthesis I (meso-diaminopimelate containing)
queuosine biosynthesis
preQ0 biosynthesis
pyruvate fermentation to isobutanol (engineered)
adenosine ribonucleotides de novo biosynthesis
guanosine ribonucleotides de novo biosynthesis
superpathway of guanosine nucleotides de novo biosynthesis I
CDP-diacylglycerol biosynthesis II
L-valine biosynthesis
0.0012
0.0010
0.0008
0.0006
T2D
HC
Lachnospiraceae bacterium_5_1_63FAA
coenzyme A biosynthesis II (mammalian)
L-lysine biosynthesis VI
pyruvate fermentation to isobutanol (engineered)
guanosine ribonucleotides de novo biosynthesis
L-valine biosynthesis
0.000025
0.000020
0.000015
0.000010
T2D
HC
Lachnospiraceae bacterium_5_1_63FAA
chorismate biosynthesis I
superpathway of L-aspartate and L-asparagine biosynthesis
coenzyme A biosynthesis II (mammalian)
coenzyme A biosynthesis I
adenosylcobalamin salvage from cobinamide I
superpathway of aromatic amino acid biosynthesis
dTDP-L-rhamnose biosynthesis I
glycogen biosynthesis I (from ADP-D-Glucose)
methylerythritol phosphate pathway I
pentose phosphate pathway (non-oxidative branch)
phosphopantothenate biosynthesis I
L-lysine biosynthesis VI
pyruvate fermentation to acetate and lactate II
L-isoleucine biosynthesis IV
sucrose degradation IV (sucrose phosphorylase)
GDP-mannose biosynthesis
CDP-diacylglycerol biosynthesis I
urate biosynthesis/inosine 5'-phosphate degradation
5-aminoimidazole ribonucleotide biosynthesis I
5-aminoimidazole ribonucleotide biosynthesis II
S-adenosyl-L-methionine cycle I
chorismate biosynthesis from 3-dehydroquinate
superpathway of 5-aminoimidazole ribonucleotide biosynthesis
galactose degradation I (Leloir pathway)
stachyose degradation
guanosine nucleotides degradation III
starch degradation V
superpathway of pyrimidine nucleobases salvage
adenosine ribonucleotides de novo biosynthesis
guanosine ribonucleotides de novo biosynthesis
thiamin formation from pyrithiamine and oxythiamine (yeast)
purine ribonucleosides degradation
CDP-diacylglycerol biosynthesis II
D-galactose degradation V (Leloir pathway)
NAD biosynthesis I (from aspartate)
adenosine nucleotides degradation II
superpathway of L-serine and glycine biosynthesis I
L-tryptophan biosynthesis
L-valine biosynthesis
0.0015
0.0010
0.0005
T2D
HC
Roseburia intestinalis
chorismate biosynthesis I
coenzyme A biosynthesis II (mammalian)
superpathway of aromatic amino acid biosynthesis
methylerythritol phosphate pathway I
phosphopantothenate biosynthesis I
peptidoglycan biosynthesis I (meso-diaminopimelate containing)
L-lysine biosynthesis III
L-lysine biosynthesis VI
tetrapyrrole biosynthesis I (from glutamate)
CDP-diacylglycerol biosynthesis I
UMP biosynthesis
S-adenosyl-L-methionine cycle I
chorismate biosynthesis from 3-dehydroquinate
UDP-N-acetylmuramoyl-pentapeptide biosynthesis II (lysine-containing)
UDP-N-acetylmuramoyl-pentapeptide biosynthesis I (meso-diaminopimelate containing)
starch degradation V
pyruvate fermentation to isobutanol (engineered)
adenosine ribonucleotides de novo biosynthesis
guanosine ribonucleotides de novo biosynthesis
purine ribonucleosides degradation
CDP-diacylglycerol biosynthesis II
L-valine biosynthesis
0.0020
0.0015
0.0010
T2D
HC
Ruminococcus sp_5_1_39BFAA

Supplementary Figure 2 Heatmaps of metabolic pathways related to differential species between T2D and HC groups in GM metagenome.
Abbreviations: T2D; type 2 diabetes; HC, healthy control.

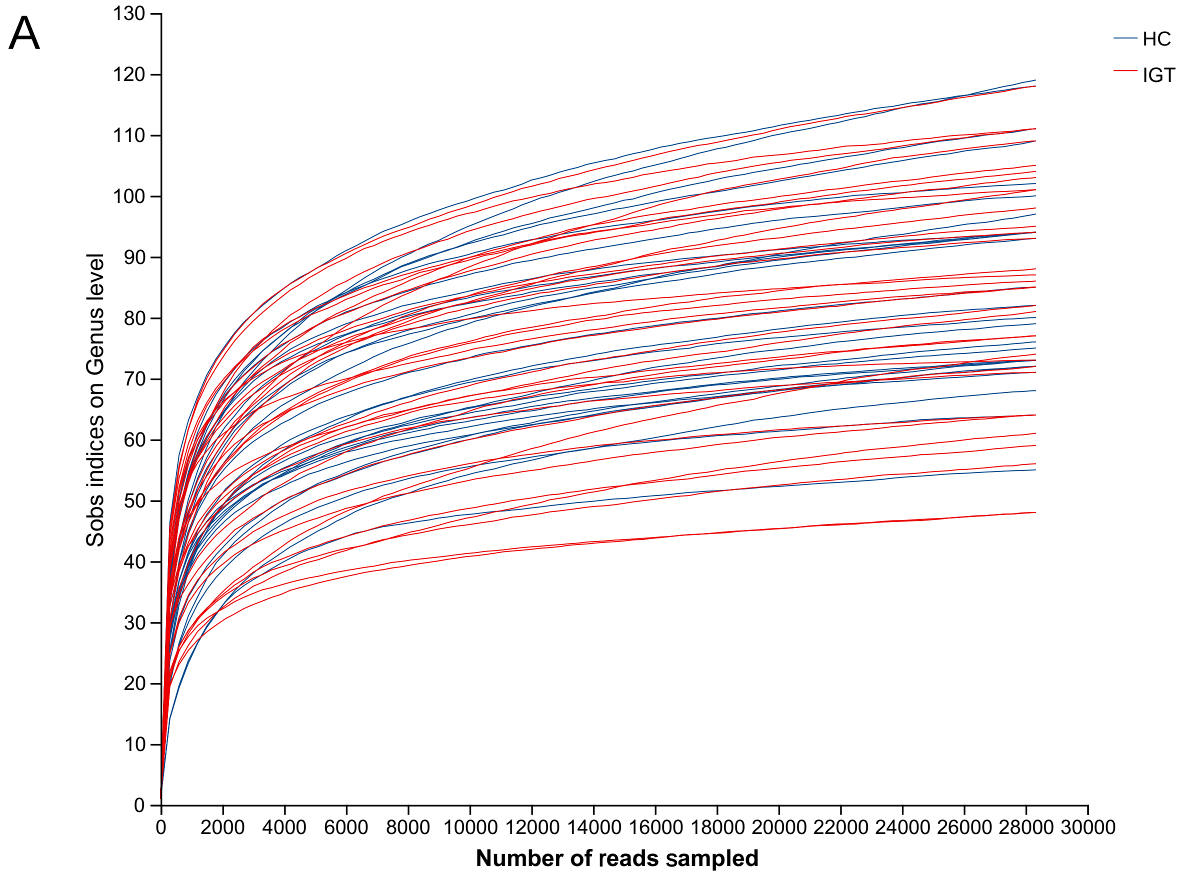

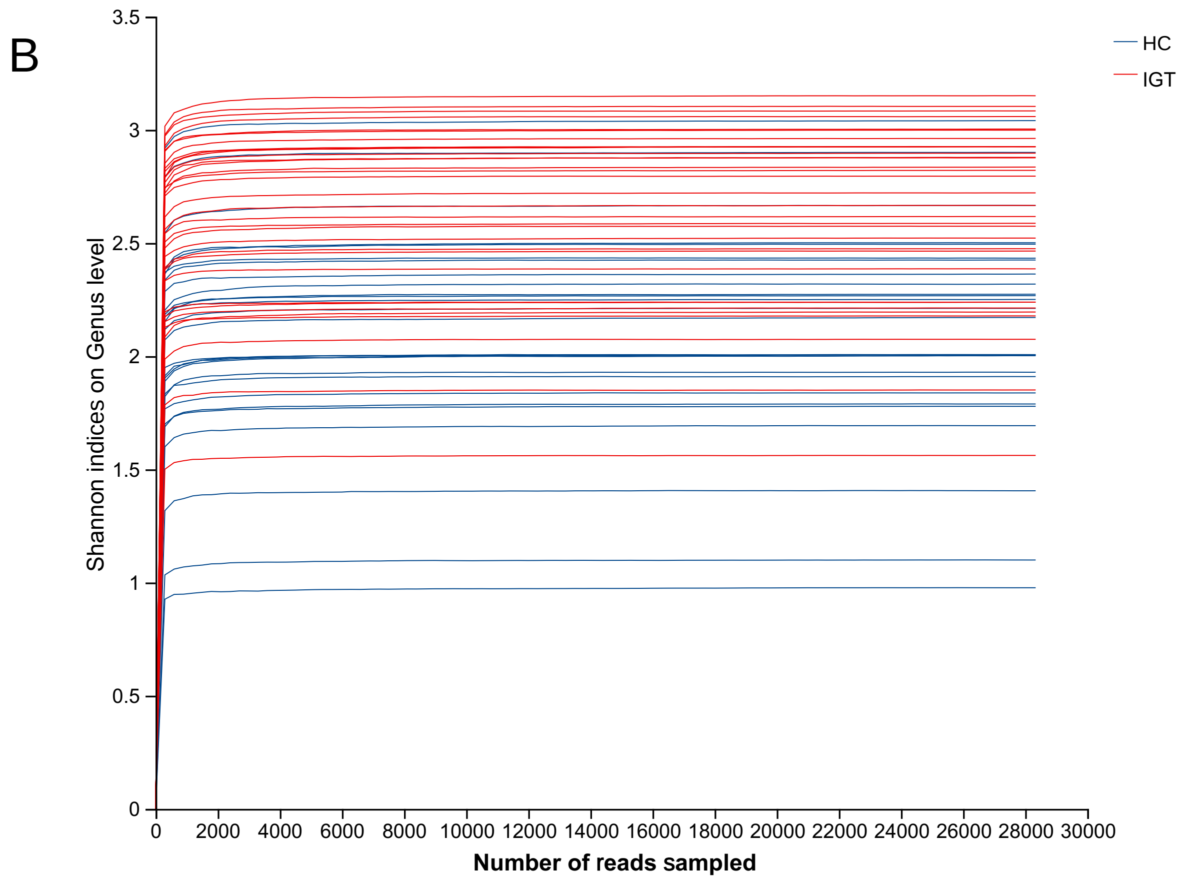

Supplementary Figure 3 The rarefaction curves illustrated that the sequencing depth was enough. The rarefaction curves of (A) Sobs indexes and (B) Shannon indexes on genus level. **Abbreviations:** IGT, impaired glucose tolerance; HC, healthy control.

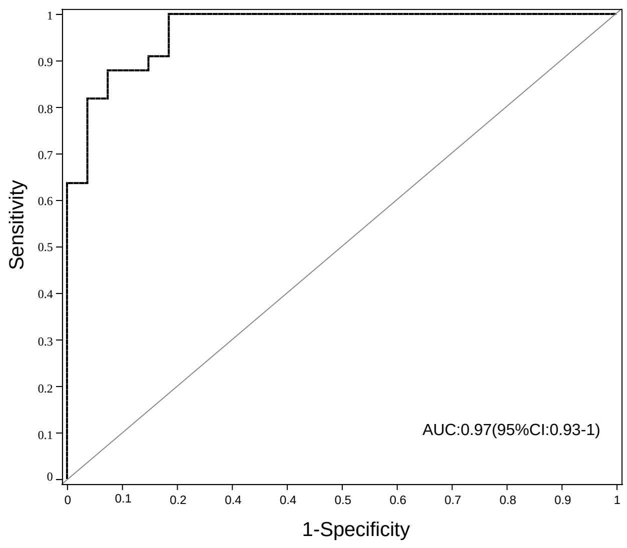

Supplementary Figure 4 The ROC curve based on the key differential genera.
Abbreviations: ROC, receiver operating characteristic; AUC, area under curve.

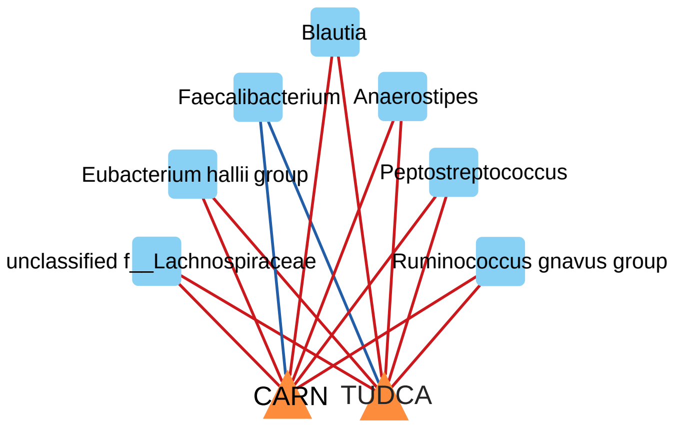

Supplementary Figure 5 The Spearman's correlations between TUDCA/CARN and the related differential genera. Blue line represents positive correlation while red line represents negative correlation.
Abbreviations: TUDCA, tauroursodeoxycholic acid; CARN, carnosine.

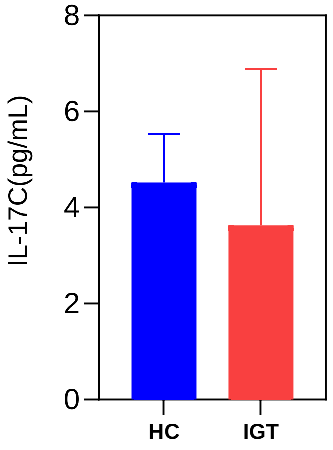

Supplementary Figure 6 The concentration of IL-17C in the peripheral plasma didn't show any difference between IGT and HC groups in the ELISA.
Abbreviations: IGT, impaired glucose tolerance; HC, healthy control; IL-17, interleukin 17; ELISA; enzyme-linked immunosorbent assay.
